# Multimodal Phytochemical and Micronutrient Profiling of *Onosma onur-koyuncui* Sezer Seeds: Antioxidant and Enzyme Activity Inhibitory Potential

**DOI:** 10.3390/antiox15101327

**Published:** 2026-10-14

**Authors:** Kübra Öğüt, Ana M. Troncoso, María del Carmen García-Parrilla, Gülmira Özek, Okan Sezer, Temel Özek

**Affiliations:** 1 Department of Pharmacognosy, Faculty of Pharmacy, Anadolu University, Eskişehir 26470, Türkiye; gozek@anadolu.edu.tr (G.Ö.); tozek@anadolu.edu.tr (T.Ö.); 2 Departamento de Nutrición y Bromatología, Toxicología y Medicina Legal, Facultad de Farmacia, Universidad de Sevilla, C/P García González No. 2, 41012 Sevilla, Spain; amtroncoso@us.es (A.M.T.); mcparrilla@us.es (M.d.C.G.-P.); 3 Department of Biology, Faculty of Science, Eskişehir Osmangazi University, Eskişehir 26040, Türkiye; oksezer@ogu.edu.tr

**Keywords:** *Onosma onur-koyuncui*, Boraginaceae, seeds, phytochemical profiling, UHPLC-HRMS/MS, GC-FID/MS, ICP-OES, antioxidant activity, α-amylase inhibition, extraction polarity

## Abstract

This study aimed to provide the first comprehensive seed-based phytochemical and biological investigation of *Onosma onur-koyuncui* Sezer, a species endemic to Türkiye, by characterizing its chemical composition and evaluating its in vitro antioxidant and enzyme inhibitory potential. A multi-technique approach combining SPME-GC-FID/MS, GC-FID/MS after silylation, ICP-OES, and UHPLC-HRMS/MS was applied to *n*-hexane and 70% aqueous methanol seed extracts, together with total phenolic and flavonoid determinations, complementary antioxidant assays, and α-amylase inhibition. *p*-Cymene (15.00%) was the major volatile, octadecatrienoic acid (27.77%) predominated in the fatty acid profile, and calcium (28.50 mg/g_dw_) was the most abundant mineral. UHPLC-HRMS/MS revealed chlorogenic acid (51.29 mg/g_dw_) and diosmetin (42.95 mg/g_dw_) as major constituents of the 70% aqueous methanol extract. This extract showed the highest phenolic and flavonoid contents and notable antioxidant activity, particularly in the DPPH assay (IC_50_ = 0.043 mg/mL), exceeding that of BHA (IC_50_ = 0.118 mg/mL), and exhibited α-amylase inhibition (IC_50_ = 0.40 mg/mL). These findings provide the first integrated characterization of *O. onur-koyuncui* seeds and highlight their phytochemical diversity and antioxidant potential.

## 1. Introduction

The genus *Onosma* L. (Boraginaceae) has approximately 250 species and is predominantly found in the Mediterranean region and Western Asia [1,2]. Numerous *Onosma* species have been widely examined for their phytochemical components and biological properties, with particular emphasis on their aerial parts and roots [3,4,5,6,7]. These investigations have identified various classes of phytochemical constituents, including naphthoquinones, flavonoids, phenolic acids, and fatty acids, which are frequently linked to significant antioxidant, antibacterial, anti-inflammatory, and enzyme inhibitory effects [8,9,10,11].

Despite the increasing interest in the pharmaceutical potential of *Onosma* species, a substantial gap persists in the scientific understanding of the seed components of this genus. The seeds serve as a significant source of bioactive chemicals, minerals, and essential fatty acids that may enhance the nutritional and medicinal value of the plants [12,13,14,15]; however, research on *Onosma* seeds remains insufficient. The phytochemical composition, biological activity, and mineral content of *Onosma* seeds have not been extensively explored. Furthermore, at present, no species of the genus *Onosma* is included in the catalog of herbal substances formally acknowledged by the European Medicines Agency (EMA) through its Committee for Herbal Medicinal Products [16].

At the family level, seeds of several Boraginaceae species have attracted attention because of their richness in polyunsaturated fatty acids and phenolic constituents associated with notable biological activities [17,18]. For instance, seed oils of *Borago officinalis* are well known for their high γ-linolenic acid content, while genera such as *Echium*, *Alkanna*, and Buglossoides have been reported as valuable sources of nutritionally important ω-3 and ω-6 fatty acids [19,20]. Moreover, seed extracts from several Boraginaceae species have demonstrated considerable antioxidant potential in ABTS and DPPH assays [18], in some cases comparable to well-known γ-linolenic acid–producing oilseeds [20]. In addition to antioxidant effects, methanolic and aqueous seed extracts of certain taxa (e.g., *Cerinthe major* and *Lithodora zahnii*) have shown dose-dependent antiproliferative activity against HT-29 colorectal cancer cells [18]. These bioactivities are frequently associated with the presence of phenolic constituents, particularly rosmarinic acid and related derivatives, which have been reported at relatively high levels in Boraginaceae seeds [21]. Collectively, these findings highlight the biological relevance of Boraginaceae seeds and support further investigation of underexplored taxa within the family.

To date, no studies have investigated the seed phytochemical content of *O. onur-koyuncui* Sezer [22], an endemic species recently identified in Türkiye. Despite the growing body of literature on *Onosma* species, it remains unclear whether the seeds of these taxa possess a comparable phytochemical richness and biological potential to other extensively studied plant parts. In particular, the relationship between the chemical composition of *Onosma* seeds and their antioxidant and enzyme inhibitory properties has not yet been systematically explored. Addressing this gap is essential to determine whether these underutilized plant materials represent a significant source of bioactive compounds. The present study aimed to conduct a comprehensive investigation of the seeds of *O. onur-koyuncui*, encompassing phytochemical profiling via UHPLC-HRMS/MS and GC-FID/MS following silylation, analysis of mineral composition, assessment of fatty acid content, and evaluation of antioxidant and enzyme inhibitory activities. Considering the extensive documentation of phenolic acids and flavonoids as contributors to antioxidant and enzyme inhibition mechanisms, correlating the phytochemical richness of the seeds with their biological potential is of significant scientific interest [23]. Accordingly, the present study investigated both the chemical profile and selected biological activities of the seeds. To achieve a broader evaluation of antioxidant properties, six widely recognized assays were conducted, including DPPH, ABTS, CUPRAC, FRAP, ORAC, and β-carotene bleaching. These complementary methods capture different aspects of antioxidant action—such as radical scavenging, electron transfer capacity, lipid peroxidation prevention, and hydrogen atom donation—enabling evaluation of different antioxidant response mechanisms [24,25,26]. Moreover, eco-friendly extraction techniques, including water-bath-assisted ultrasonic extraction and solid-phase microextraction (SPME) were employed to reduce solvent consumption and environmental repercussions [27]. The findings from this study are expected to improve current knowledge regarding the phytochemical and pharmacological attributes of *Onosma* species and to provide a basis for future nutraceutical or pharmaceutical investigations.

In this field of sustainable natural product research, the valorization of underutilized plant parts, such as seeds, demonstrates an attractive approach in accordance with green chemistry principles [28]. Seeds are frequently regarded as agricultural or botanical byproducts; however, they may contain bioactive compounds of pharmaceutical significance. Investigating these neglected resources fosters scientific advancement and promotes environmental sustainability [29]. For this purpose, the present study provides a detailed investigation of the seeds of *O. onur-koyuncui*, particularly in terms of their phytochemical composition and biological activity. Furthermore, it represents the first comprehensive phytochemical and biological investigation of seeds within the genus *Onosma*.

## 2. Materials and Methods

### 2.1. Chemicals and Reagents

*n*-Hexane, methanol, ethanol, dimethyl sulfoxide (DMSO), glacial acetic acid, N,O-bis(trimethylsilyl)trifluoroacetamide (BSTFA, containing 1% TMCS), hydrochloric acid, α-amylase from porcine pancreas (Type VI-B, ≥10 units/mg solid), acarbose, and β-carotene were obtained from Sigma-Aldrich (St. Louis, MO, USA). AAPH, ABTS, Trolox, BHA, DPPH, Folin–Ciocalteu reagent, aluminum chloride, fluorescein, linoleic acid, gallic acid, quercetin, and the remaining analytical-grade reagents used in the spectrophotometric assays were obtained from Merck (Darmstadt, Germany). Reference standards of phenolic acids and flavonoids used for UHPLC-HRMS/MS identification and quantification were obtained from Sigma-Aldrich or Merck. All solvents and reagents were of analytical or chromatographic grade, as appropriate.

### 2.2. Instruments and Analytical Equipment

GC-FID/MS analysis was performed using an Agilent 5975 GC-MSD system (Agilent Technologies, Santa Clara, CA, USA). UHPLC-HRMS/MS analysis was performed using a Thermo Scientific™ Vanquish™ Flex UHPLC system in conjunction with an Orbitrap Exploris™ 120 high-resolution mass spectrometer (Thermo Fisher Scientific™, Bremen, Germany). Data were acquired and processed using Xcalibur software (version 3.0.63, Thermo Fisher Scientific, Waltham, MA, USA). Absorbance measurements were performed using a BioTek PowerWave XS microplate reader (BioTek Instruments, Winooski, VT, USA). A 65 µm polydimethylsiloxane/divinylbenzene (PDMS/DVB) fiber (Supelco, Bellefonte, PA, USA) was used for SPME analysis. Lyophilization was performed using a Christ Alpha 1-2 LDplus freeze dryer (Martin Christ, Osterode am Harz, Germany), and ultrasonic-assisted extraction was carried out using a Bandelin SONOREX RK 510 H water-bath sonicator (Bandelin, Berlin, Germany).

### 2.3. Plant Material

**Figure 1:**
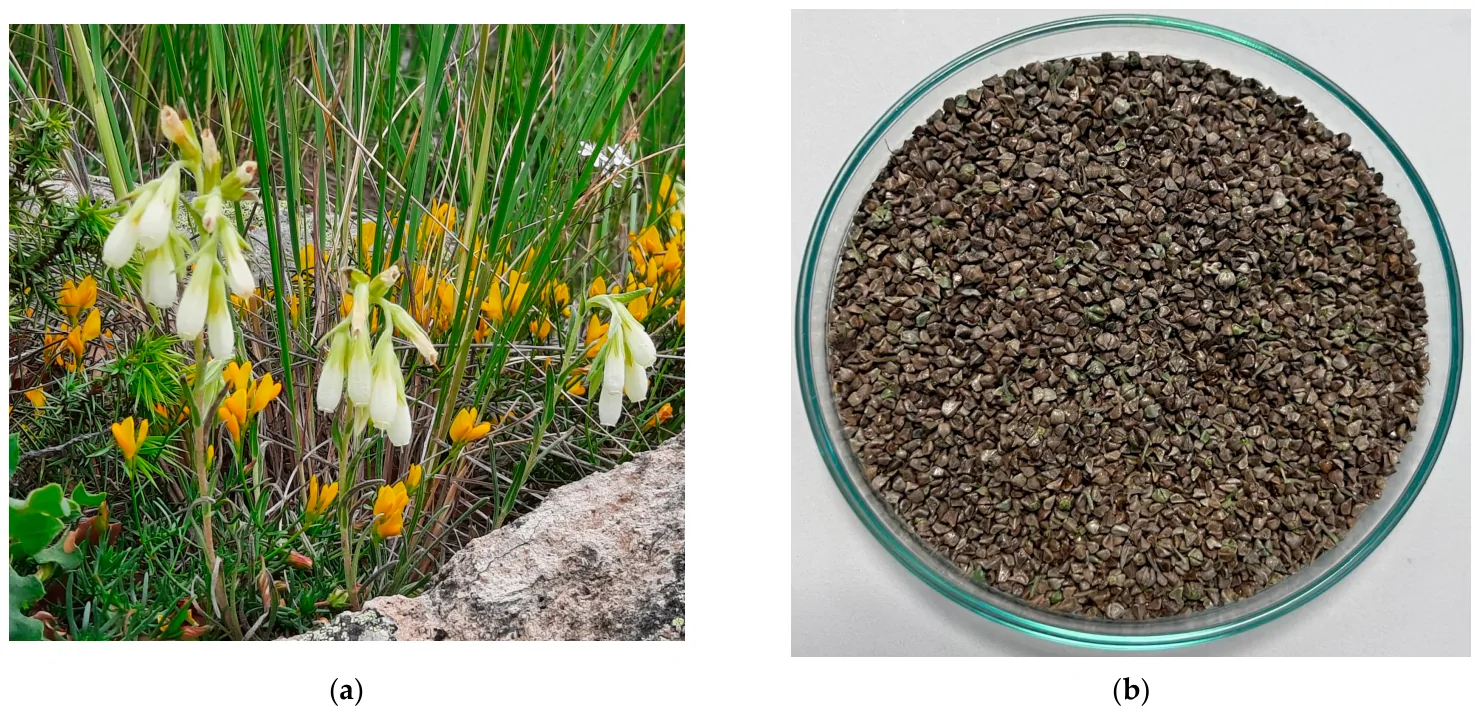
Morphological features and plant material of *O. onur-koyuncui* used in the study (**a**) General view of the aerial parts of *O. onur-koyuncui* during the flowering stage in its natural habitat in Kütahya Province, Türkiye. (**b**) Dried seeds of *O. onur-koyuncui* used for extraction, fatty acid and mineral analyses, SPME-based volatile profiling, and biological activity assays.

Seeds of *O. onur-koyuncui* were collected at the time of seed maturation on 28 July 2023 from the region of Sökmen village, Kütahya Province, Türkiye (39°35′15″ N, 30°11′43″ E; 1050 m a.s.l.) during a botanical survey. The plant material was collected from its natural habitat and was not obtained from a commercial supplier or store. The plant material was dried in the shade at ambient temperature (approximately 25 °C) with continuous air circulation to preserve thermolabile components. Taxonomic identification was performed by Assoc. Prof. Okan Sezer (Eskişehir Osmangazi University, Faculty of Science, Department of Biology), and a voucher specimen was deposited at the Herbarium of Anadolu University, Faculty of Pharmacy (Voucher No. 15902). Following the drying process, the seeds were carefully separated manually from the dried plant material to ensure sample purity and prevent contamination with other plant parts. The separated seeds were subsequently used for extraction and analytical investigations (Figure 1). The collection procedure complied with the relevant legal and ethical regulations governing wild plant sampling in Türkiye.

The seeds were collected at full maturity, as indicated by the change in color from green to dark brown and the formation of a hardened seed structure. Mature seeds measured approximately 2.1–2.6 mm in length and 1.7–2.0 mm in width, exhibited a smooth and glossy surface, and possessed a distinct beak-shaped apex. No reticulate ornamentation or spiny structures were observed on the seed surface. Only fully developed, uniformly colored, and morphologically intact seeds were selected for further analyses.

### 2.4. Extraction Procedures

#### 2.4.1. Lipid Extraction and Fatty Acid Derivatization

Lipid extraction and fatty acid methylation were performed prior to GC-FID/MS analysis according to established protocols [30] with minor modifications. Briefly, powdered seeds (0.15 g) were extracted with 3.0 mL chloroform–methanol (2:1, *v*/*v*) using a commercial lipid extraction kit. Following phase separation and filtration, the lipid fraction was dried under a nitrogen stream. For fatty acid methyl ester (FAME) preparation, the dried lipid extract was treated with 1.0 mL boron trifluoride–methanol solution and 0.3 mL *n*-hexane and heated at 95 °C for 1 h. After cooling, *n*-hexane and distilled water were added, and the mixture was centrifuged at 500× *g* for 5 min. The upper *n*-hexane phase containing the FAMEs was collected, dried under nitrogen, and analyzed by GC-FID/MS.

#### 2.4.2. Microsteam Distillation-Solid-Phase Microextraction (MSD-SPME)

Powdered seed material (1.0 g) was mixed with 3.0 mL of distilled water in a 25 mL flask and subjected to microsteam distillation using a Claisen distillation head and reflux condenser. Volatile compounds were extracted using a 65 μm PDMS/DVB SPME fiber, preconditioned at 250 °C for 15 min. Following equilibration at the boiling temperature of the system, simultaneous distillation and headspace extraction were performed for 3.0 min. The extracted volatiles were subsequently thermally desorbed in the GC injection port and analyzed by GC-FID/MS according to the established method [31].

#### 2.4.3. General Extraction of Plant Material

The powdered seeds (20 g) were extracted by maceration using *n*-hexane (24 h, five cycles) and 70% aqueous methanol (24 h, seven cycles) on a shaker at room temperature. Following each maceration, the mixtures were subjected to ultrasonic-assisted extraction for 30 min in a water bath (40 kHz, 100 W), while the temperature was monitored and maintained at 25 ± 2 °C. Prior to the second ultrasonic treatment, the mixtures were stirred for 1 h, followed by an additional 30 min ultrasonic extraction under the same conditions. After completion of the *n*-hexane extraction, the plant material was air-dried to remove residual solvent and subsequently extracted with 70% aqueous methanol under the same maceration and ultrasonic-assisted extraction conditions. The supernatants were filtered through Whatman No. 1 paper. Organic solvents were removed using a rotary evaporator (Heidolph Instruments GmbH & Co. KG, Schwabach, Germany) at temperatures below 40 °C under reduced pressure. The aqueous residues were subjected to lyophilization in a freeze dryer. All dried extracts were stored in amber glass vials at 4 °C until analysis.

#### 2.4.4. Preparation of Silyl Derivatives of Extracts

The *n*-hexane and aqueous methanol (70:30, *v*/*v*) extracts of *O. onur-koyuncui* seeds were silylated prior to GC-FID/MS analysis according to [32]. For silylation, 2 mg of the dried extract was dissolved in 80 μL of pyridine and mixed with 80 μL of BSTFA containing 1% TMCS. The mixture was heated at 90 °C for 1 h, after cooling, 1.0 μL was injected into the GC-FID/MS system for analysis.

### 2.5. Analytical Methods

Seeds and their *n*-hexane and 70% aqueous methanol extracts were analyzed using complementary chromatographic and spectrometric techniques, as described below.

#### 2.5.1. Gas Chromatography-Flame Ionization Detection/Mass Spectrometry Analysis (GC-FID/MS)

Volatile compounds obtained by MSD-SPME and fatty acid methyl esters (FAMEs) were analyzed by GC-FID/MS according to previously reported conditions [33]. Separation was performed on an Agilent Innowax FSC column (60 m × 0.25 mm, 0.25 μm film thickness) using helium at 0.8 mL/min. The oven temperature was maintained at 60 °C for 10 min, increased to 220 °C at 4 °C/min and held for 10 min, and then increased to 240 °C at 1 °C/min. The injector temperature was 250 °C with a split ratio of 40:1, and the FID temperature was 300 °C. Mass spectra were acquired in EI mode at 70 eV over *m*/*z* 35–450. Compounds were identified by comparison with authentic standards, relative retention indices (RRIs) calculated using C_8_–C_40_ *n*-alkanes, and spectral data from the Wiley-NIST, MassFinder, Adams, and in-house libraries. Relative abundances were calculated from FID peak areas.

The *n*-hexane and 70% aqueous methanol extracts, silylated as described in Section 2.4.4, were analyzed by GC-FID/MS using an Agilent 7890A gas chromatograph coupled to a 5975C MSD. Separation was achieved on an HP-5 FSC column (30 m × 0.25 mm, 0.25 μm film thickness) with helium at 0.8 mL/min. The oven temperature was increased from 100 to 320 °C at 3 °C/min and held at 320 °C for 16.67 min. Injections were performed in splitless mode, with injector and FID temperatures of 250 and 300 °C, respectively. Mass spectra were acquired in EI mode at 70 eV over *m*/*z* 35–1050. Compounds were identified using the Wiley GC/MS Library, the in-house Special Özek Silyl Derivative Library, reported RRI values, and literature mass spectral data [32]. Relative abundances were calculated from FID peak areas [34].

#### 2.5.2. UHPLC-HRMS/MS Orbitrap Phenolic Compound Analysis

Phenolic compounds in the *n*-hexane and 70% aqueous methanol extracts were analyzed by UHPLC-HRMS/MS. Chromatographic separation was performed on a Waters Acquity BEH C18 column (100 mm × 2.1 mm i.d., 1.7 µm) at 40 °C and a flow rate of 0.5 mL/min. The mobile phases were water (A) and methanol containing 0.1% formic acid (B), with the following gradient: 5% B (0–1 min), 5–100% B (1–10 min), 100% B (10–12 min), followed by re-equilibration to 5% B. The injection volume was 5 µL.

Mass spectrometric analysis was performed in negative ESI mode using Top5 data-dependent acquisition, with full-scan and MS/MS resolutions of 70,000 and 17,500, respectively. The source parameters were spray voltage, −3.0 kV; capillary temperature, 320 °C; probe heater temperature, 400 °C; S-lens RF level, 50; sheath gas, 60; and auxiliary gas, 25 arbitrary units. Data were processed using TraceFinder 5.1. Compounds were identified by comparison with 87 authentic reference standards based on retention time, accurate mass (≤5 ppm), isotope pattern, and MS/MS fragmentation, with isotope-pattern matching scores >80% considered acceptable [35]. Compound identification was performed using authentic reference standards based on matching retention times, accurate mass measurements, and MS/MS fragmentation patterns. Δmass (ppm) values were calculated relative to the theoretical precursor ions ([M−H]^−^). All identified compounds were annotated based on accurate mass measurements, retention times, MS/MS fragmentation patterns, and comparison with authentic reference standards.

#### 2.5.3. Mineral Analysis by ICP-OES

For mineral analysis, the powdered seeds (0.5 g) were subjected to digestion with concentrated nitric acid using a microwave digestion system. The digested samples were diluted with ultrapure water and analyzed using Inductively Coupled Plasma Optical Emission Spectrometry (ICP-OES; PerkinElmer, Waltham, MA, USA) for simultaneous multielement detection. The operating conditions were set as follows: RF power of 1.2 kW, plasma gas flow rate of 15 L/min, auxiliary gas flow rate of 1.5 L/min, and nebulizer gas flow rate of 0.8 L/min. The elements quantified included Mg, Na, Al, Zn, Ti, Ni, Fe, Cu, Ca, and K. Each element was quantified at its specified emission wavelength: Mg (280.271 nm), Na (589.592 nm), Al (396.153 nm), Zn (206.200 nm), Ti (334.940 nm), Ni (221.648 nm), Fe (238.204 nm), Cu (324.752 nm), Ca (393.366 nm), and K (766.490 nm). Calibration curves were established using certified multi-element standard solutions at different concentration levels. The results were expressed as milligrams of element per gram of dried plant material (mg/g_dw_). All analyses were performed in compliance with the ISO 11885 protocol [36,37].

### 2.6. Determination of Total Phenolic and Flavonoid Contents

The total phenolic content (TPC) was determined using Folin–Ciocalteu reagent (FCR) [38]. Extracts (5 mg/mL) were reacted with Folin–Ciocalteu reagent and 20% (*w*/*v*) sodium carbonate, incubated for 2 h at 25 °C in the dark, and measured at 760 nm. Gallic acid was used for calibration (0.01–1.0 mg/mL), and results were expressed as mg gallic acid equivalents GAE/g_extract_.

The total flavonoid content (TFC) was determined using the AlCl_3_ colorimetric method [31]. Extracts (5 mg/mL) were reacted with AlCl_3_ and incubated for 40 min in the dark before absorbance measurement at 415 nm. Quercetin was used for calibration (0.01–1.0 mg/mL), and results were expressed as mg quercetin equivalents QE/g_extract_. All experiments were carried out in triplicate, and results were reported as mean ± standard deviation (SD).

### 2.7. Evaluation of Biological Activities

#### 2.7.1. Free Radical Scavenging Effect (DPPH)

The free radical scavenging activity was determined using a modified Brand-Williams method [39]. Extracts (2 mg/mL) were incubated with DPPH solution (0.08 mg/mL in methanol) for 30 min in the dark, and absorbance was measured at 517 nm. Gallic acid (0.1 mg/mL) was used as the positive control. Radical scavenging activity was expressed as percentage inhibition, and IC_50_ values were determined by nonlinear regression (SigmaPlot v15.0) for samples reaching >50% inhibition. Measurements were performed in triplicate and reported as mean ± SD.

#### 2.7.2. Ferric Reducing Antioxidant Power (FRAP) Assay

The ferric reducing antioxidant power (FRAP) of extracts were determined according to the established method [40]. Freshly prepared FRAP reagent consisted of acetate buffer (300 mM, pH 3.6), TPTZ (10 mM in 40 mM HCl), and FeCl_3_·6H_2_O (20 mM) at a 10:1:1 ratio. Extracts (1 mg/mL in methanol containing 10% DMSO) were reacted with the FRAP reagent for 8 min at room temperature, and absorbance was measured at 593 nm. Antioxidant capacity was calculated using a Trolox calibration curve and expressed as µmoltrolox/mg_extract_. All experiments were performed in triplicate, and results were reported as mean ± standard deviation (SD).

#### 2.7.3. Oxygen Radical Absorbance Capacity (ORAC) Assay

The oxygen radical absorbance capacity (ORAC) assay was performed with minor modifications [41]. Samples or Trolox standards were incubated with fluorescein (45 nM) and AAPH (15 mM) at 37 °C, and fluorescence was recorded every minute for 60 min at excitation/emission wavelengths of 485/528 nm. ORAC values were calculated from the area under the fluorescence decay curve (AUC) using a Trolox calibration curve (0.5–9.5 μM) and expressed as µmol Trolox/mg extract. Measurements were performed in quadruplicate and reported as mean ± SD.

#### 2.7.4. Cupric Ion Reducing Antioxidant Capacity (CUPRAC) Assay

The CUPRAC assay was performed according to a previously described method [42] with minor modifications. Extracts (5 mg/mL in methanol containing 10% DMSO) were reacted with CuCl_2_ (1.0 × 10^−2^ M), neocuproine (7.5 × 10^−3^ M), and ammonium acetate buffer (1.0 M, pH 7.0). After incubation at 25 °C for 30 min, absorbance was measured at 450 nm. Antioxidant capacity was calculated using a Trolox calibration curve and expressed as µmol Trolox/mg extract. Measurements were performed in quadruplicate and reported as mean ± SD.

#### 2.7.5. Trolox Equivalent Antioxidant Capacity (TEAC) Assay

The TEAC assay was performed using the ABTS^+•^ radical cation decolorization assay and expressed as Trolox equivalent antioxidant capacity [43]. Extracts were prepared in methanol containing 10% DMSO and reacted with freshly prepared ABTS^•+^ solution adjusted to an absorbance of 0.700–0.800 at 734 nm. After incubation for 30 min in the dark, absorbance was measured at 734 nm. Antioxidant capacity was calculated using a Trolox calibration curve and expressed as μmol Trolox/mg_extract_. Measurements were performed in triplicate and reported as mean ± SD.

#### 2.7.6. Lipid Peroxidation Inhibition (β-Carotene Bleaching) Assay

The lipid peroxidation inhibitory activity of the extracts was evaluated using a modified β-carotene bleaching assay [44]. The β-carotene/linoleic acid emulsion was prepared using β-carotene (1 mg/mL in chloroform), Tween 20 (200 mg), linoleic acid (25 μL), and oxygenated ultrapure water (50 mL) after evaporation of chloroform. Extracts and BHA, used as the positive control, were prepared in methanol containing 10% DMSO. Absorbance was recorded at 492 nm every 15 min for 105 min at 50 °C. Antioxidant activity was calculated as the percentage inhibition of β-carotene bleaching relative to the control. Measurements were performed in triplicate and reported as mean ± SD.

#### 2.7.7. α-Amylase Inhibitory Activity Assay

The α-amylase inhibitory activity assay was performed with minor modifications to the previously reported I_2_/KI-based method [44,45]. The positive control was acarbose. Extracts (5 mg/mL in methanol containing 10% DMSO) were serially diluted and incubated with α-amylase (0.8 U/mL in 20 mM sodium phosphate buffer, pH 6.9) at 37 °C for 10 min. Starch solution (0.05%) was then added, followed by a further 10 min incubation at 37 °C. The reaction was terminated with 1 M HCl, and freshly prepared I_2_/KI reagent was added before measuring absorbance at 630 nm. Appropriate enzyme-free, inhibitor-free, and sample blanks were included. IC_50_ values were determined by nonlinear regression for samples reaching 50% inhibition. Measurements were performed in triplicate and reported as mean ± SD.

### 2.8. Statistical Evaluation of Bioactivity Assays

Statistical analyses were performed using one-way analysis of variance (ANOVA) followed by Tukey’s post hoc test, with statistical significance set at *p* < 0.05. IC_50_ values for antioxidant and α-amylase inhibitory activities were calculated using SigmaPlot v15.0. Results are presented as mean ± standard deviation (SD).

## 3. Results and Discussions

### 3.1. Chemical Composition of Fatty Acids

**Figure 2:**
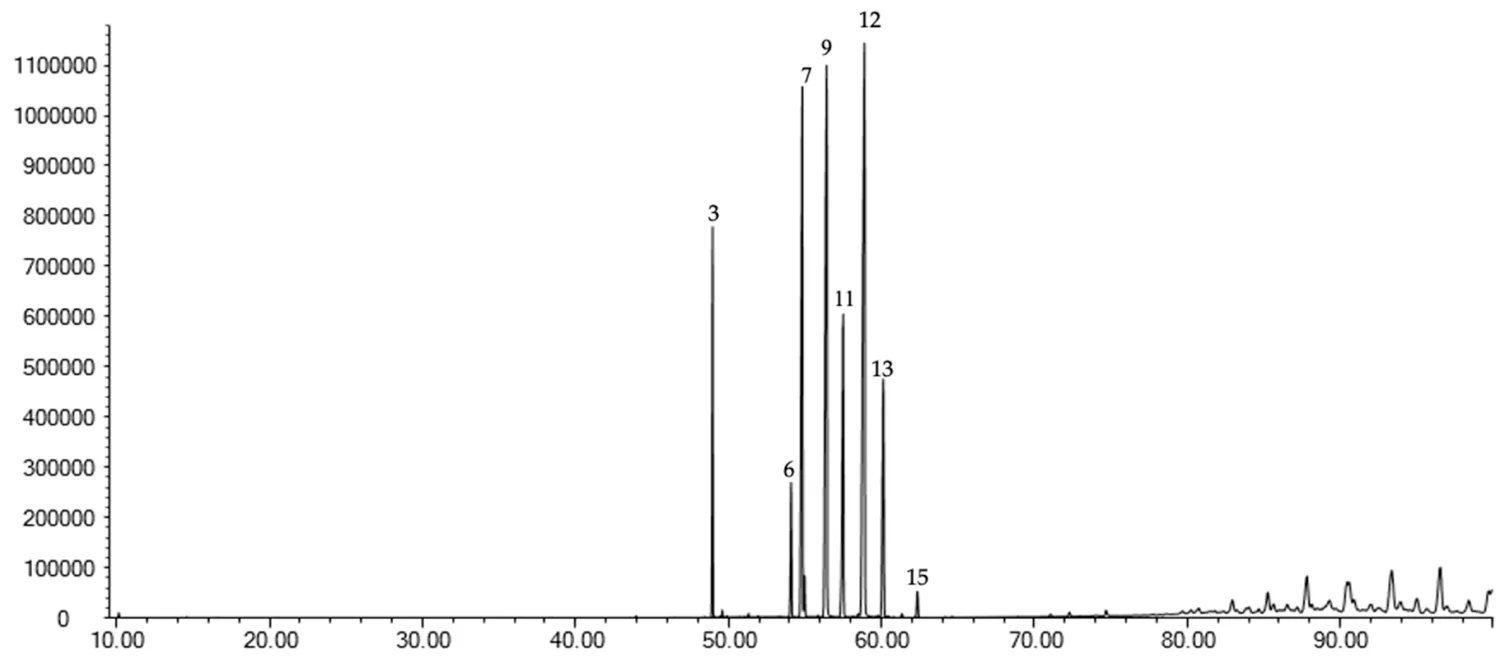
GC FID/MS chromatogram of fatty acid methyl esters obtained from *O. onur koyuncui* seeds. The numbered peaks correspond to the compound numbers listed in Table 1.

The present study provides the first comprehensive characterization of the fatty acid composition of *O. onur-koyuncui* seeds. The fatty acid composition of *O. onur-koyuncui* seeds was analyzed through microscale lipid extraction followed by transesterification, enabling the GC-FID/MS-based analysis of fatty acid methyl esters (FAMEs). FAMEs were identified by comparison of mass spectral data with commercial libraries and confirmed by co-injection with authentic standards when available. The GC-FID/MS analytical results are presented as mean values accompanied by their respective standard deviations. The detected FAMEs are listed in Table 1, along with their retention times and relative abundances, while Figure 2 illustrates the corresponding GC-FID/MS chromatogram obtained from *O. onur-koyuncui* seed extract.

In the present investigation, 20 different fatty acids were identified and quantified in the seeds of *O. onur-koyuncui* using the GC-FID/MS analysis. The findings demonstrated that the seed fatty acid profile was completely characterized, accounting for 100% of the total detected fatty acid composition, with the extract especially abundant in ω-3 fatty acids compared with ω-6, ω-7, and ω-9. The principal compounds identified were octadecatrienoic acid (27.77 ± 0.08%), linoleic (23.01 ± 0.13%), and oleic (18.24 ± 0.07%) acids. The high proportion of polyunsaturated fatty acids, especially ω-3 derivatives, is noteworthy, as these compounds are widely associated with antioxidant, anti-inflammatory, and enzyme-modulating activities [46]. In addition, ω-3 fatty acids are known to participate in oxidative stress regulation, membrane stabilization, and enzyme-mediated signaling pathways, and may contribute to antioxidant defense systems through redox-associated biochemical processes [47,48]. Therefore, the predominance of these fatty acids may be associated with the significant antioxidant and α-amylase inhibitory activities observed in the present study [49,50]. Accordingly, the comprehensive fatty acid profile provides a phytochemical basis for the in vitro bioactivities observed in this study and offers meaningful chemotaxonomic insight, further supporting the nutritional relevance of *O. onur-koyuncui* seeds. From a broader taxonomic perspective, seeds of several Boraginaceae genera have been reported to contain substantial levels of polyunsaturated fatty acids [21,51]. For instance, *Echium* species may contain approximately 37% α-linolenic acid together with ~10% stearidonic acid and ~10% γ-linolenic acid [19,51], while seeds typically accumulate 15–24% γ-linolenic acid [52,53]. Similarly, *Symphytum* [54,55,56,57] up to 23–28% γ-linolenic acid [18,58], and *Anchusa* taxa generally exhibit 11–22% γ-linolenic acid in their seed oils [59,60]. In this context, the fatty acid composition of *O. onur-koyuncui* seeds supports the view that members of the Boraginaceae family may represent valuable sources of nutritionally relevant polyunsaturated fatty acids.

To the best of our knowledge, this study provides the first comprehensive and quantitative characterization of the seed fatty acid profile within the genus *Onosma*. Previous reports on fatty acids in the genus have primarily relied on essential oil analyses or indirect detection methods, without a focused lipidomic evaluation. Therefore, the present work provides a detailed and systematic insight into seed oil composition of *O. onur-koyuncui*, contributing both to the phytochemical knowledge of the genus and to its potential valorization as a source of bioactive lipids. Beyond their nutritional relevance, the predominance of polyunsaturated fatty acids, particularly ω-3 derivatives, provides important insight into the biological behavior of the extracts [61,62]. It is well established that ω-3 and ω-6 fatty acids contribute to antioxidant mechanisms by modulating lipid peroxidation processes and interacting with reactive oxygen species [63,64]. In this context, the high proportion of α-linolenic and related fatty acids in *O. onur-koyuncui* seeds may play a key role in stabilizing oxidative systems, which is consistent with the strong antioxidant responses observed in multiple assays employed in this study. Moreover, the coexistence of these fatty acids with phenolic compounds detected in the extracts suggests a potential synergistic interaction, where lipid-soluble and polar antioxidants may act through complementary mechanisms [65,66]. Such combined effects are increasingly recognized as a critical factor in enhancing the overall bioactivity of plant extracts, rather than the action of individual constituents alone [67]. Therefore, the fatty acid composition not only reflects the nutritional profile of the seeds but also supports their functional role in the observed in vitro biological activities [54,55,56,57].

### 3.2. Chemical Composition of Volatile Compounds

**Figure 3:**
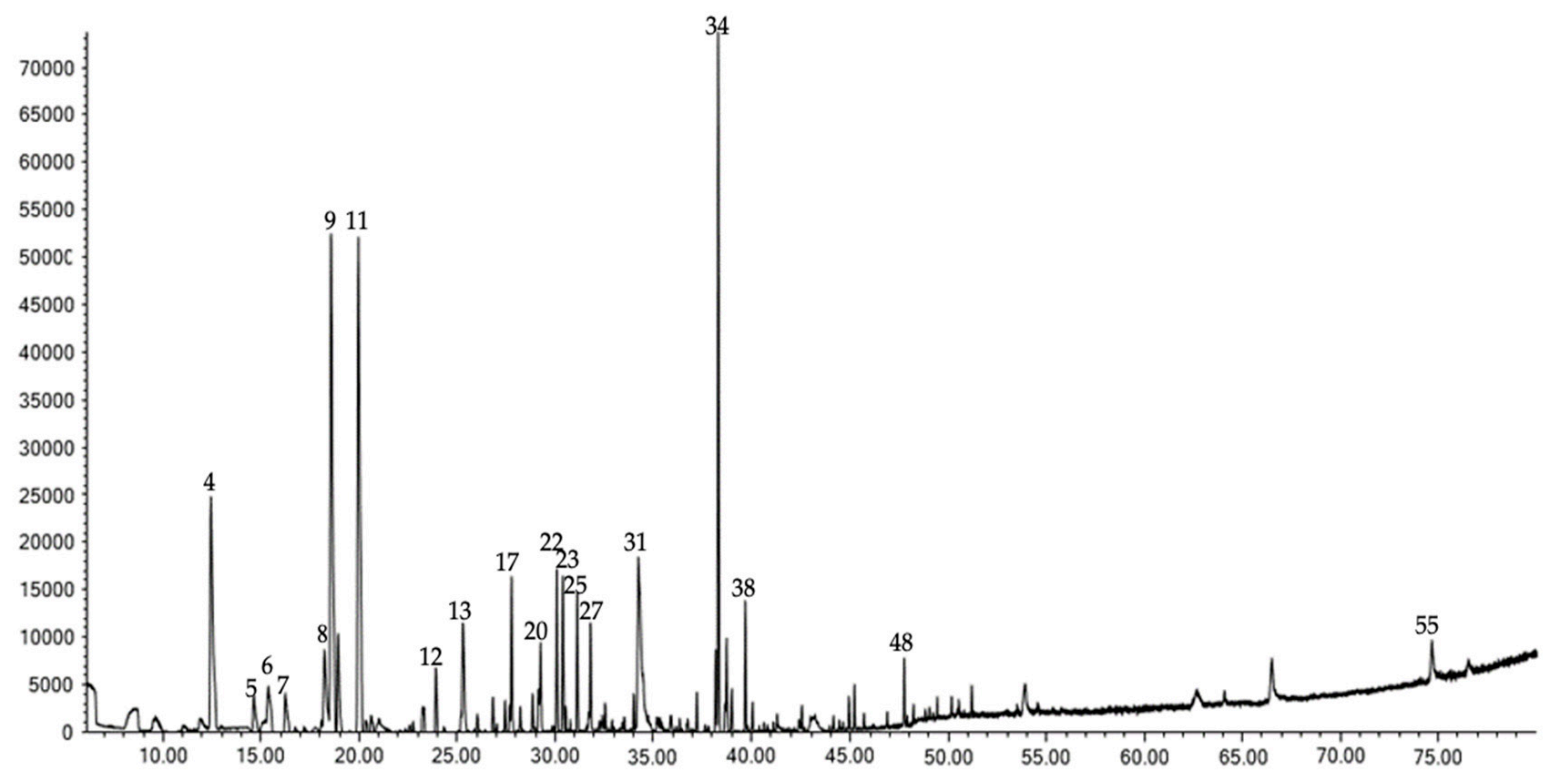
GC FID/MS chromatogram of volatile compounds obtained by MSD-SPME from *O. onur-koyuncui* seeds. Peak numbers in the chromatogram correspond to the compound numbers listed in Table 2.

The volatile components of the seeds of *O. onur-koyuncui* were investigated using microsteam distillation-solid-phase microextraction (MSD-SPME) in conjunction with GC-FID/MS. Replicated measurements were performed for the volatile analysis. The detected compounds are listed in Table 2 according to their elution sequence on the HP-Innowax FSC column, together with their retention indices (RRI), identification methods, and relative percentages, while the corresponding GC-FIS/MS chromatogram obtained by MSD-SPME is presented in Figure 3.

This study used solid-phase microextraction (SPME) instead of essential oil distillation to examine the volatile components in the seeds of *O. onur-koyuncui*. The endemic and geographically restricted nature of plant material throughout the investigation is essential. SPME is a rapid, chemical-free, and highly sensitive technique commonly employed to analyze plant material and evaluate volatile compounds from botanical sources. Its suitability for small sample sizes makes it especially advantageous in phytochemical investigations of rare or endemic species. Literature demonstrates that SPME efficiently extracts plant material volatiles from delicate plant tissues and minimizes sample deterioration. Unlike conventional methods, SPME enables the efficient profiling of volatile compounds as it preserves the structural integrity of the plant material. The method has remarkable consistency and sensitivity, making it ideal for accurate quantification, even with minimal botanical samples. This study’s choice of SPME demonstrates a strategic method for enhancing analytical efficiency and conserving valuable plant material. This method enabled the efficient identification of volatile compounds in the seeds of *O. onur-koyuncui* while avoiding material loss—an essential consideration for studying uncommon, endemic species.

In the present study, a total of 55 volatile compounds were identified in the seeds of *O. onur-koyuncui* by GC-FID/MS analysis. The major compounds found were γ-terpinene (15.15 ± 0.03%), *p*-cymene (15.00 ± 0.26%), cuminaldehyde (9.62 ± 0.07%), and benzene acetaldehyde (8.44 ± 0.09%). The predominance of γ-terpinene, *p*-cymene, and cuminaldehyde in the volatile profile is particularly noteworthy, as these compounds are biosynthetically related and commonly co-occur in plant volatile matrices, supporting the chemically interconnected nature of the volatile profile. Such a composition may be associated with the protective chemical profile of the seeds, as previously been reported in relation to environmental stress tolerance and antimicrobial-related activities. In addition, oxygenated aromatic constituents such as cuminaldehyde and benzene acetaldehyde may contribute not only to the biological activity but also to the characteristic aroma profile of the seeds. The coexistence of terpenoid and aromatic compounds suggests a chemically diverse volatile matrix, which may enhance ecological functionality and contribute to the overall bioactive potential of the plant material, including antioxidant and antimicrobial effects reported for similar compounds in previous studies [100,101,102]. In addition, several of the predominant volatile constituents identified in the present study, including γ-terpinene, *p*-cymene, and cuminaldehyde, have previously been associated with antioxidant, antimicrobial, and enzyme-modulating properties in different plant systems. Therefore, the volatile profile of *O. onur-koyuncui* seeds may represent a chemically relevant component contributing to the overall phytochemical complexity of the extracts. The volatile profile observed in the seeds provides valuable chemical insight into the potential biological behavior of this plant organ. Although the bioassays were conducted on solvent-derived extracts, certain volatile constituents—particularly those with moderate lipophilicity—may partially contribute to the observed activities. In addition, the presence of these compounds may indicate a chemically interconnected phytochemical profile that also includes non-volatile bioactive constituents. Therefore, the volatile composition may be considered an indirect indicator of the overall phytochemical potential of the seeds [103,104,105]. The volatile composition observed in this study demonstrates a distinct profile compared to the aerial and root parts previously reported for other *Onosma* species, highlighting clear organ-specific chemical differentiation within the genus. To the best of our knowledge, this is the first report describing the volatile constituents of *O. onur-koyuncui* seeds using MSD-SPME, and more broadly, one of the first datasets providing a detailed characterization of seed volatiles within the genus *Onosma*. This finding contributes novel insight into the seed chemistry of the genus and expands the current understanding of its phytochemical diversity.

In the literature, aerial parts of *O. isaurica* Boiss. & Heldr. have been reported to contain hexahydrofarnesyl acetone (20.3%), phytol (19.0%), farnesyl acetone (8.1%), and neophytadiene isomer tetradecanal (7.0%) as the major volatile constituents [106]. Similarly, aerial parts of *O. bulbotricha* DC. were characterized by hexahydrofarnesyl acetone (11.6%), farnesyl acetone (9.9%), hexadecanal (8.8%), *(E)-*geranyl acetone (7.4%), and neophytadiene isomer I (7.3%) as dominant components [106]. In the roots *of O. sieheana* Hayek, *p*-cymene (27.32%) was reported as the principal volatile compound [107]. In contrast to these previous reports on aerial parts and roots, the present study provides the first volatile profile of the seeds, revealing a distinct composition and further highlighting the chemical diversity within the genus. This variation in volatile composition may reflect organ-specific phytochemical differences among plant tissues, since seeds are generally associated with storage and reproductive functions, whereas aerial parts and roots are more commonly related to environmental interactions such as herbivore deterrence and stress adaptation [108,109,110]. Consequently, the distinct chemical profile observed in the seeds further emphasizes the organ-specific phytochemical diversity reported within the genus [111,112]. These findings emphasize the importance of organ-targeted phytochemical investigations and demonstrate that seeds represent a previously underexplored yet chemically unique component of *Onosma* species.

### 3.3. Mineral Analysis

The mineral composition of *O. onur-koyuncui* seeds was analyzed using inductively coupled plasma optical emission spectrometry (ICP-OES). The concentrations of Mg, Na, Al, Zn, Ti, Ni, Fe, Cu, Ca, and K were determined and are presented in Table 3 as mg/g_dw_.

Of all the minerals studied, Ca had the highest concentration, followed by K and Mg. Relatively elevated concentrations of Ca, K, and Mg were observed in the seeds of *O. onur-koyuncui.* These levels may contribute to the species’ nutritional value and potential health advantages. When expressed on a dry weight basis, the Ca content of *O. onur-koyuncui* seeds (28.50 mg/g dw; ≈2850 mg/100 g) indicates a highly concentrated mineral profile. For comparison, commonly consumed Ca sources such as dairy products (e.g., milk, yogurt, cheese) [113,114,115] and plant-derived foods (e.g., leafy vegetables and legumes) typically provide markedly lower Ca amounts on a fresh weight basis [116,117,118]. However, the direct nutritional relevance of these findings should be interpreted cautiously, considering differences in serving size, moisture content, and the absence of bioavailability data. Previous reports indicate that members of the Boraginaceae family may exhibit a notable capacity for calcium accumulation, although most available data are derived from aerial parts or roots rather than seeds. For instance, comparatively high Ca levels have been reported in Boraginaceae species such as *O. bracteatum* leaves (103.97 mg/g) and *O. echioides* roots (91.16 mg/g). However, substantial organ-dependent variation has also been documented within the family; for example, the fruit of *Cordia latifolia* contained markedly lower Ca levels (6.38 mg/g) [119]. Also, the literature revealed that the primary minerals in the roots of *O. sericea* Boiss. were Ca, and K, followed by Mg, Na, and Fe [120]. In the roots and aerial parts of *O. gmelinii*’s Ledeb, Ca and K were identified as the major minerals, accompanied by lower levels of Mg, Na, and Fe [121]. These crucial macroelements are essential for bone health, cardiovascular functioning, and enzymatic activity [122,123,124,125]. In addition, mineral composition may also contribute to the overall biochemical characteristics of the seeds, since elements such as Mg, Ca, and Fe have been associated with enzyme-related functions and redox-associated processes in biological systems [125,126,127,128]. These findings suggest that while Boraginaceae taxa may possess an inherent capacity for Ca enrichment, the specific plant organ plays a critical role in determining mineral abundance [119,129]. In this context, the present study provides the first insight into the Ca content and overall mineral composition of *O. onur-koyuncui* seeds, thereby addressing an important gap in the knowledge of seed mineral profiles within the genus *Onosma*.

### 3.4. Extraction Yields

This research presents the first examination of the phytochemical profile and bioactivity of *O. onur-koyuncui* seeds. Consequently, these investigations have contributed to bridging the knowledge gap about *O. onur-koyuncui* within the scientific field. The seeds of *O. onur-koyuncui* were extracted using *n*-hexane and 70% aqueous methanol to obtain both apolar and polar phytochemical constituents. The extract yields are presented in Table 4.

The *n*-hexane extract was obtained as a brownish-beige, viscous liquid, whereas the 70% aqueous methanol extract was obtained as a tacky, dark brown solid. Under the extraction conditions employed, the *n*-hexane extract exhibited a higher yield than the 70% aqueous methanol extract. This finding is consistent with the substantial amount of lipophilic constituents detected in the seeds, including unsaturated fatty acids. However, because the two solvents differ markedly in polarity, their extraction yields should not be interpreted as a direct measure of the relative abundance of polar and nonpolar constituents in the seed matrix. Rather, the two extraction systems provided complementary fractions for the characterization of chemically distinct groups of seed constituents.

### 3.5. Chemical Composition of Silylated Extracts

**Figure 4:**
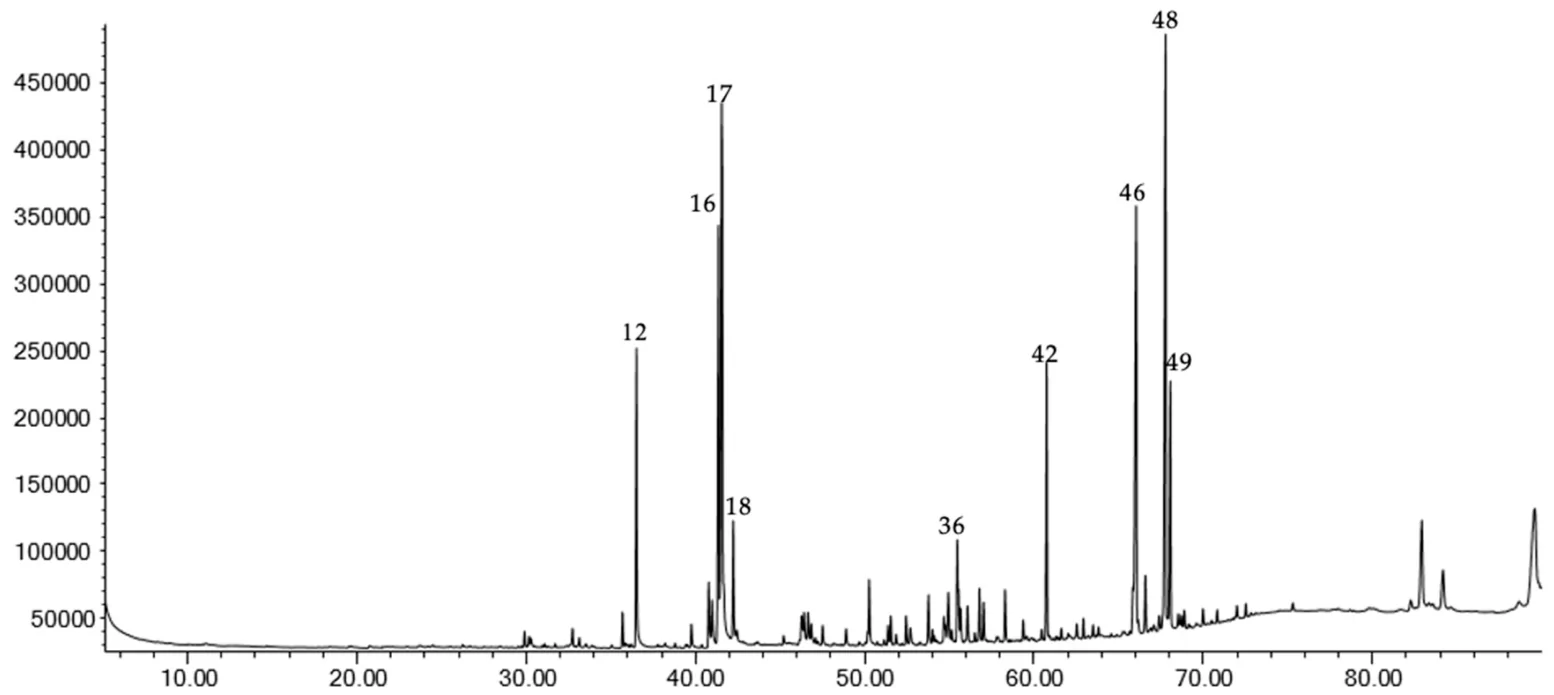
GC-FID/MS chromatogram of the silylated *n*-hexane extract of *O. onur-koyuncui* seeds. The numbered peaks correspond to the compound numbers listed in Table 5.

**Figure 5:**
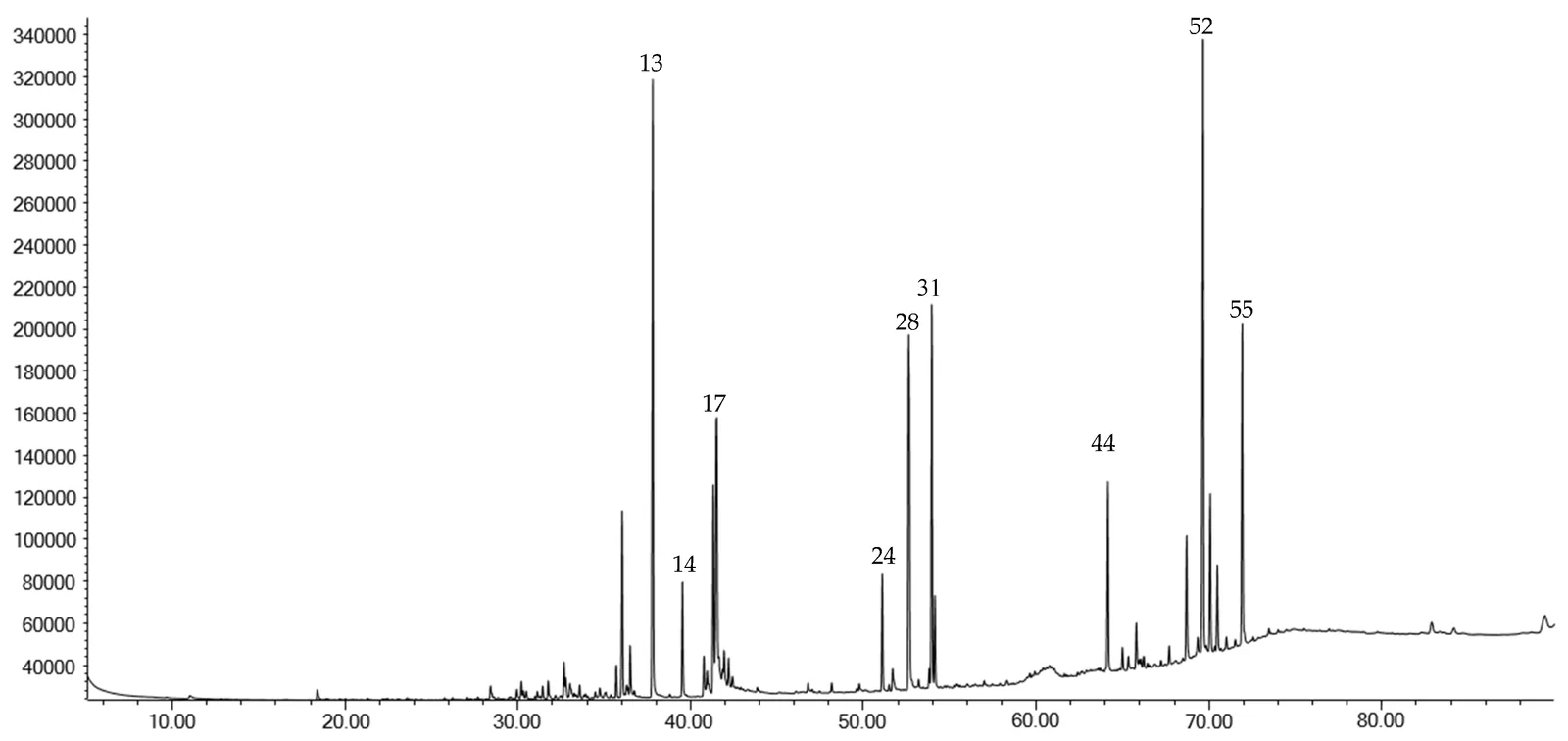
GC-FID/MS chromatogram of the silylated 70% aqueous methanol extract of *O. onur-koyuncui* seeds. The numbered peaks correspond to the compound numbers listed in Table 5.

The seed extracts (*n*-hexane and 70% aqueous methanol extracts) of *O. onur-koyuncui* were derivatized with BSTFA to enable the gas chromatographic analysis of silylated components. The resulting silyl derivatives were separated using a non-polar HP-5 capillary column, using a programmed temperature gradient to improve chromatographic separation. A series of standard *n*-alkanes (C_8_–C_40_) was used to determine the relative retention indices (RRI) of the constituents. The amount of the substances was measured using the total area of the peaks from the GC-FID data, without normalization. The representative chromatograms of the respective extracts are shown in Figure 4 and Figure 5. The identification profile of the derivatized *n*-hexane and 70% aqueous methanol extract of *O. onur-koyuncui* seeds is presented in Table 5.

GC-MS profiling of structurally varied phytochemicals in *O. onur-koyuncui* seeds has benefited from silylation. This method is effective for molecules that have active hydrogen groups, like hydroxyls, carboxyls, and amines, which are challenging to study using GC-MS as a result of their low volatility or thermal instability [130,131,132]. These functional groups form trimethylsilyl derivatives, which improve their volatility and thermal stability, making them easier to separate and quantify [133,134,135]. Silylation was especially useful for detection of non-volatile polar compounds such as sugars, sugar alcohols, and hydroxycinnamic acids [136,137]. Derivatization is a strategic method for determining the variety of phytochemical composition in plant matrices [138,139]. This approach expanded the detectable phytochemical range and provided a broarder characterization of phytochemical composition of *O. onur-koyuncui* seeds, contributing to the phytochemical knowledge of both species and the *Onosma* genus.

The silylation-based GC-FID/MS analysis of *n*-hexane and 70% aqueous methanol extracts of *O. onur-koyuncui* allowed the identification of a wide range of phytochemicals. The results demonstrate that trimethylsilyl derivatization is useful for detection of chemically diverse phytochemical constituents, including sterols, fatty acids, and sugars, which are not readily detectable by conventional GC-MS analysis without derivatization. The *n*-hexane extract revealed *(Z)-*9-octadecenoic acid (14.86 ± 0.06%) and β-sitosterol (13.69 ± 0.13%). On the other hand, the 70% aqueous methanol extract showed that rosmarinic acid (14.03 ± 0.17%) and catechol lactate (12.90 ± 0.11%) were the main components. The major constituents identified in the seed extracts belong to chemical classes that have been extensively associated with biological functions in the literature. Unsaturated fatty acids and phytosterols have been reported to exhibit antioxidant and membrane-associated properties [140,141], whereas phenolic compounds such as rosmarinic acid and catechol derivatives are commonly associated with redox-relating and enzyme-relating activities [142]. Accordingly, the phytochemical composition of the seed extracts can indicate a biochemical potential for biological activity. The simultaneous occurrence of lipophilic and phenolic compounds may be associated with complementary interactions within the extracts [143].

Previous phytochemical investigations of *Onosma* species using GC-MS remain limited. The analysis of the *O. rutila* Hub.-Mor. ethanol extract revealed twelve predominant components, and the primary compounds found were 9,12-octadecadienoic acid (15.90%), palmitic acid (13.74%), 9-eicosene (11.14%), and 3,5-di-tert-butylbromobenzene (9.83%) [4]. GC/MS analysis of *O. sintenisii* Hausskn. ex Bornm. showed that the main components in the methanol extracts are retronecine (13.94%), α-D-glucopyranosiduronic acid (10.86%), melaniline (7.5%), and 1,2-butanediol (4.02%) [6]. The observed differences between the *n*-hexane and 70% aqueous methanol extracts clearly reflect polarity-dependent distribution of phytochemical constituents, resulting in distinct chemical profiles for each fraction [144]. The predominance of fatty acids, sterols, and lipophilic compounds in the *n*-hexane extract, alongside the enrichment of phenolic acids, sugars, and polar phytochemical constituents in the 70% aqueous methanol extract, demonstrates a well-defined chemical separation based on solvent selectivity [145,146]. This complementary distribution of pytochemical constituents highlights the importance of employing solvents with differing polarities to achieve a more comprehensive phytochemical characterization [147]. In this context, the combined analytical strategy applied in the present study provides a broader and more integrated understanding of the chemical composition of *O. onur-koyuncui* seeds.

### 3.6. Phenolic Compound Profile by UHPLC-HRMS/MS

In the present investigation, UHPLC-HRMS/MS was used to perform both qualitative and quantitative studies of the phenolic compounds in the *n*-hexane and 70% aqueous methanol extracts of *O. onur-koyuncui* seeds. Compound identification was performed using authentic reference standards based on matching retention times, accurate mass measurements, and MS/MS fragmentation patterns. Quantitative determination was carried out using external calibration curves constructed from authentic standards. Table 6 and Table 7 summarize the identified and quantified compounds. Representative chromatograms of the *n*-hexane and 70% aqueous methanol extracts are provided in the Frontiers Supplementary Material (Supplementary Figures S1 and S2) to support peak identification and compound distribution. In addition, extracted ion chromatograms (EICs) and corresponding MS/MS (MS^2^) spectra of the phenolic compounds identified and quantified in Table 6 and Table 7 are presented in Supplementary Figures S3 and S4. Calibration curves for all quantified standards, including their regression equations and coefficients of determination (R^2^), are provided in Supplementary Table S5, supporting the reliability and accuracy of the quantitative analysis. Additional analytical datasets and repository-associated files are publicly available through the Zenodo repository (https://doi.org/10.5281/zenodo.20271343).

The UHPLC-HRMS/MS analysis of the *n*-hexane extract revealed a relatively limited phenolic profile compared to the 70% aqueous methanol extract. Among the identified compounds, epicatechin (0.64 ± 0.00 mg/g_dw_) was detected as the predominant phenolic constituent, followed by diosmetin (0.44 ± 0.02 mg/g_dw_), whereas quercetin, taxifolin, and naringenin were present at lower concentrations. Among the phenolic compounds identified in the *n*-hexane extract, epicatechin, diosmetin, naringenin, quercetin, and taxifolin were characterized based on their retention times, accurate mass measurements, and MS/MS fragmentation patterns in comparison with authentic reference standards. Epicatechin (RT: 4.33 min) exhibited a deprotonated molecular ion [M−H]^−^ at *m*/*z* 289.07175, corresponding to the molecular formula C_15_H_14_O_6_. The MS/MS spectrum showed characteristic fragment ions at *m*/*z* 245.0132, 205.0366, and 179.0111, consistent with the typical fragmentation behavior of flavan-3-ols [148,149]. Taxifolin (RT: 5.01 min) exhibited a deprotonated molecular ion [M−H]^−^ at *m*/*z* 303.05093, corresponding to the molecular formula C_15_H_12_O_7_. The MS/MS spectrum showed fragment ions at *m*/*z* 285.04086, 175.04005, and 151.00378. The fragment ion at *m*/*z* 285 was consistent with the loss of H_2_O, while the ion at *m*/*z* 151 may be associated with characteristic flavonoid ring fragmentation, supporting the structural assignment of taxifolin [150,151]. Quercetin (RT: 6.65 min) exhibited a deprotonated molecular ion [M−H]^−^ at *m*/*z* 301.03506, corresponding to the molecular formula C_15_H_10_O_7_. The MS/MS spectrum showed characteristic fragment ions at *m*/*z* 151.00386 and 178.99893. The fragment ion at *m*/*z* 151 is commonly associated with RDA cleavage of the flavonoid C-ring, supporting the structural assignment of quercetin [152]. Naringenin (RT: 6.79 min) exhibited a deprotonated molecular ion [M−H]^−^ at *m*/*z* 271.06137, corresponding to the molecular formula C_15_H_12_O_5_. The MS/MS spectrum showed characteristic fragment ions at *m*/*z* 151.00385 and 119.05033. The fragment ion at *m*/*z* 151 is associated with flavanone C-ring fragmentation, supporting the structural assignment of naringenin [153,154]. Diosmetin (RT: 7.36 min) exhibited a deprotonated molecular ion [M−H]^−^ at *m*/*z* 299.05588, corresponding to the molecular formula C_16_H_12_O_6_. The MS/MS spectrum showed characteristic fragment ions at *m*/*z* 284.033 and 151.003. The fragment ion at *m*/*z* 284 may be associated with the loss of a methyl group, while the ion at *m*/*z* 151 is consistent with characteristic flavonoid ring fragmentation, supporting the structural assignment of diosmetin [155,156]. These fragmentation features, together with retention time and accurate mass matching with authentic reference standards, supported the structural assignment of the identified phenolic compounds in the *n*-hexane extract. In contrast to the *n-*hexane extract, the 70% aqueous methanol extract exhibited a richer and more diverse phenolic composition. Chlorogenic acid (51.29 ± 0.77 mg/g_dw_) and diosmetin (42.95 ± 1.69 mg/g_dw_) were identified as the predominant constituents, followed by taxifolin, 4-hydroxybenzoic acid, quercetin, caffeic acid, and protocatechuic acid. Protocatechuic acid (RT: 2.12 min) exhibited a deprotonated molecular ion [M−H]^−^ at *m*/*z* 153.0192, corresponding to the molecular formula C_7_H_6_O_4_. The MS/MS spectrum showed characteristic fragment ions at *m*/*z* 109.0293 and 108.0170. The fragment ion at *m*/*z* 109 may be attributed to the loss of CO_2_ (44 Da) from the carboxyl group, which is a characteristic fragmentation pathway commonly reported for hydroxybenzoic acid derivatives in negative ESI mode, supporting the structural assignment of protocatechuic acid [157]. 4-Hydroxybenzoic acid (RT: 3.26 min) exhibited a deprotonated molecular ion [M−H]^−^ at *m*/*z* 137.02435, corresponding to the molecular formula C_7_H_6_O_3_. The MS/MS spectrum showed characteristic fragment ions at *m*/*z* 93.03473 and 65.0396. The fragment ion at *m*/*z* 93 may be associated with decarboxylation (loss of CO_2_) from the precursor ion, which is commonly observed in the fragmentation behavior of hydroxybenzoic acid derivatives in negative ESI mode, supporting the structural assignment of 4-hydroxybenzoic acid. Similarly, salicylic acid (2-hydroxybenzoic acid) (RT: 5.87 min) exhibited a deprotonated molecular ion [M−H]^−^ at *m*/*z* 137.02431 with characteristic fragment ions at *m*/*z* 93.03461 and 65.0397, consistent with the reported fragmentation behavior of hydroxybenzoic acid derivatives involving decarboxylation pathways in negative ionization mode [158,159,160]. Chlorogenic acid (RT: 3.83 min) exhibited a deprotonated molecular ion [M−H]^−^ at *m*/*z* 353.08759, corresponding to the molecular formula C_16_H_18_O_9_. The MS/MS spectrum showed characteristic fragment ions at *m*/*z* 191.05635, 127.04014, and 93.03461. The fragment ion at *m*/*z* 191 is widely recognized as a diagnostic ion corresponding to the quinic acid moiety of caffeoylquinic acids in negative ESI mode [161]. Furthermore, the fragment ion at *m*/*z* 127 may result from further fragmentation of the quinic acid ion through the loss of H_2_O and CO_2_, while the fragment at *m*/*z* 93 is consistent with lower-mass quinic acid–derived fragments reported for chlorogenic acid derivatives [162]. These fragmentation features supported the structural assignment of chlorogenic acid. Caffeic acid (RT: 3.99 min) exhibited a deprotonated molecular ion [M−H]^−^ at *m*/*z* 179.0349, corresponding to the molecular formula C_9_H_8_O_4_. The MS/MS spectrum showed characteristic fragment ions at *m*/*z* 135.04527, 134.03746, 107.05002, and 89.03983. The fragment ion at *m*/*z* 135 is commonly associated with decarboxylation (loss of CO_2_) from the precursor ion and is considered a characteristic fragment of caffeic acid derivatives in negative ESI mode [163,164]. Taxifolin (RT: 5.02 min) exhibited a deprotonated molecular ion [M−H]^−^ at *m*/*z* 303.05109, corresponding to the molecular formula C_15_H_12_O_7_. The MS/MS spectrum showed fragment ions at *m*/*z* 285.04068, 175.04013, and 151.00366, which were highly consistent with the fragmentation pattern observed in the *n*-hexane extract and characteristic for flavanonol derivatives in negative ESI mode [163,165]. Naringin (RT: 5.80 min) exhibited a deprotonated molecular ion [M−H]^−^ at *m*/*z* 579.17188, corresponding to the molecular formula C_27_H_32_O_14_. The MS/MS spectrum showed characteristic fragment ions at *m*/*z* 271.06158, 151.00381, and 119.05031. The fragment ion at *m*/*z* 271 is associated with the formation of the naringenin aglycone following the loss of the neohesperidose moiety, which represents a characteristic fragmentation pathway of flavanone glycosides in negative ESI mode. Furthermore, the fragment ions at *m*/*z* 151 and 119 are consistent with RDA cleavage of the flavanone C-ring, supporting the structural assignment of naringin [155]. Eriodictyol (RT: 6.27 min) exhibited a deprotonated molecular ion [M−H]^−^ at *m*/*z* 287.05624, corresponding to the molecular formula C_15_H_12_O_6_. The MS/MS spectrum showed characteristic fragment ions at *m*/*z* 151.00374, 135.04526, 107.01386, 83.01392, and 65.00333. The fragment ion at *m*/*z* 151 is consistent with RDA cleavage of the flavanone C-ring and is considered a characteristic A-ring fragment of flavanone aglycones in negative ESI mode. In addition, the fragment ion at *m*/*z* 135 may be associated with complementary ring-cleavage pathways derived from the flavanone skeleton, while the lower-mass ions at *m*/*z* 107, 83, and 65 are consistent with further fragmentation of primary RDA products reported for related flavonoids [155,166]. Abscisic acid (RT: 6.53 min) exhibited a deprotonated molecular ion [M−H]^−^ at *m*/*z* 263.12872, corresponding to the molecular formula C_15_H_20_O_4_. The MS/MS spectrum showed characteristic fragment ions at *m*/*z* 219.13921, 204.11578, and 153.09222. The fragment ion at *m*/*z* 219 may be associated with CO_2_-related loss from the precursor ion, while the fragment at *m*/*z* 153 is consistent with further side-chain fragmentation of the abscisic acid skeleton reported for ABA-derived ions in negative ESI mode [167,168]. Abscisic acid is widely recognized as a major stress-responsive phytohormone involved in plant adaptation to abiotic stress conditions [169]. Quercetin and naringenin were also detected in the 70% aqueous methanol extract, exhibiting fragmentation behaviors consistent with those observed in the *n*-hexane extract and previously reported flavonoid fragmentation pathways in negative ionization mode [152,153,154]. Luteolin (RT: 6.90 min) exhibited a deprotonated molecular ion [M−H]^−^ at *m*/*z* 285.03989, corresponding to the molecular formula C_15_H_10_O_6_. The MS/MS spectrum showed characteristic fragment ions at *m*/*z* 133.02953, 65.00332, and 63.02406. The fragment ion at *m*/*z* 133 is consistent with a 1,3B^−^ RDA fragment generated by cleavage of the flavone C-ring, which is considered a characteristic fragmentation pathway of luteolin and related flavone derivatives in negative ESI mode. The lower-mass ions at *m*/*z* 65 and 63 were consistent with further fragmentation of primary RDA-derived ions reported for flavonoid aglycones [170]. Diosmetin (RT: 7.34 min) exhibited a deprotonated molecular ion [M−H]^−^ at *m*/*z* 299.05594, corresponding to the molecular formula C_16_H_12_O_6_. The MS/MS spectrum showed characteristic fragment ions at *m*/*z* 284.03284, 151.00352, and 107.0138. The fragment ion at *m*/*z* 284 may be attributed to the loss of a methyl group ([M−H−CH_3_]^−^), which is a characteristic fragmentation pathway of methoxylated flavonoids in negative ESI mode. In addition, the fragment ion at *m*/*z* 151 is consistent with RDA cleavage of the flavone C-ring, while the ion at *m*/*z* 107 may be associated with further fragmentation of primary RDA-derived ions reported for methoxylated flavonoid derivatives.

Overall, the 70% aqueous methanol extract exhibited a richer phenolic composition than the *n*-hexane extract, with chlorogenic acid (51.29 ± 0.77 mg/g_dw_) and diosmetin (42.95 ± 1.69 mg/g_dw_) identified as the predominant constituents, whereas epicatechin (0.64 ± 0.00 mg/g_dw_) was the major phenolic compound detected in the *n*-hexane extract. This study is, to our knowledge, the first phytochemical profiling of seed extracts from the *Onosma* genus, as there are no previous studies on their chemical composition. These identified compounds—widely known for their antioxidant and α-amylase inhibitory properties [171,172] may contribute to the biological activities observed in the present investigation. In the absence of prior phytochemical studies on *Onosma* seeds, comparisons have been established with the principal compounds identified in the aerial parts, flowers, and roots of different *Onosma* species.

Many different types of studies have investigated the phytochemical content of the aerial parts and roots from different *Onosma* species, with an important focus on phenolic compounds, flavonoids, and naphthoquinones [8,11,107,173,174,175,176,177,178,179,180]. Previous studies on different *Onosma* species revealed notable variations in phenolic composition depending on the plant organ and extraction solvent. In *O. bulbotricha* DC., 4-hydroxybenzoic acid was identified as the major compound, with concentrations of 1566 µg/g_extract_ in water and 9374 µg/g_extract_ in methanol extracts; vanillic acid (145 µg/g_extract_) was the predominant component in the ethyl acetate extract [5]. For *O. riedliana* Binzet & Orcan, pinoresinol was found to be the main compound, with amounts of 2992 µg/g_extract_ in ethyl acetate and 73,628 µg/g_extract_ in methanol extracts. Furthermore, 4-hydroxybenzoic acid (703.5 µg/g_extract_) was found in the ethyl acetate extract, and hesperidin (32,455.3 µg/g_extract_) was found in the methanol extract of this species [181]. Furthermore, *O. sericea* Boiss. and *O. stenoloba* Hausskn. ex Riedl contained important components such as apigenin, kaempferol, and rosmarinic acid. In *O. inexspectata* Teppner, important compounds included apigenin 7-glucoside (26.400 µg/g_extract_), chlorogenic acid (21.360 µg/g), and luteolin 7-glucoside (19.812 µg/g). The *O. armena* DC. species highlighted chlorogenic acid (36.970 µg/g_extract_) and hesperidin (248.000 µg/g_extract_) [182]. Finally, rosmarinic acid was consistently found as the predominant compound in all extracts of *O. gigantea* Lam. with concentrations of 2.65 µg/g in ethyl acetate, 296.45 µg/g_dry plant_ in methanol, and 56.40 µg/g_dry plant_ in aqueous extracts [183]. *O. siehena* Hayek root methanol extracts contain methyl palmitoleate as a major compound [182]. *Onosma* flower extracts contain phenolic acids and flavonoid glycosides, with species-specific prevailing compounds. Luteolin 7-glucoside, apigenin 7-glucoside, and rosmarinic acid are the main compounds found in *O. bourgaei* Boiss., *O. trachytricha* Boiss., *O. lycaonica* Hub.-Mor., and *O. papillosa* Riedl., according to chromatographic and mass spectrometry studies [175,182,184,185,186,187].

The phenolic composition observed in the present study demonstrates a clear organ-dependent variation when compared to previously reported aerial and root extracts of *Onosma* species. In particular, the high abundance of chlorogenic acid and diosmetin in the aqueous methanol extract suggests a strong enrichment of bioactive phenolic constituents in the seed matrix. These compounds are widely associated with antioxidant and enzyme inhibitory mechanisms, supporting the biological activity results obtained in this study [186,187]. Moreover, the simultaneous presence of flavonoids, phenolic acids, and simple phenolics indicates a chemically diverse and structurally complex phytochemical profile [188]. This compositional diversity may enhance synergistic interactions among compounds, thereby contributing to the overall bioactivity of the extracts [189,190,191]. Collectively, these findings highlight the seeds of *O. onur-koyuncui* as a previously unexplored but promising source of phenolic compounds with potential functional applications.

### 3.7. Total Phenolic, Flavonoid Contents

The *n*-hexane and 70% aqueous methanol extracts of *O. onur-koyuncui* seeds were evaluated for their total phenolic and flavonoid contents. Total phenolic content was expressed as gallic acid equivalents (mg GAE/g_extract_) and flavonoid content in quercetin equivalents. Table 8 shows total phenolic and flavonoid content.

The 70% aqueous methanol extract of *O. onur-koyuncui* seeds showed markedly higher total phenolic and flavonoid contents, with a total phenolic content of 112.19 ± 4.98 mg GAE/g_extract_ and a total flavonoid content of 51.42 ± 0.85 mg QE/g_extract_. The amount of phenolic content found in the seed extract was higher than what has been reported for the aerial parts of other *Onosma* species. Upon reviewing the studies on the total phenolic content of several *Onosma* species, available data are mainly limited for the aerial parts. Prior research on *O. bulbotricha* DC., *O. riedliana*, *O. sericea* Boiss. and *O. stenoloba* Hausskn. ex Riedl, *O. bracteosa* Hausskn. & Bornm., and *O. isaurica* Boiss. & Heldr. indicated total phenolic levels varying from 24.18 to 97.62 mg GAE/g_extract_, depending on the extraction solvent and the investigated plant component. The total flavonoid content of *Onosma* aerial parts was reported to range from 4.30 ± 0.29 mg QE/g_extract_ to 54.98  ±  0.05 mg RE/g_extract_ [11,175,181,192,193]. This study provides the first comprehensive insight into the phenolic and flavonoid content of *Onosma* seeds, with a particular focus on the phytochemical composition of *O. onur-koyuncui*.

The markedly higher total phenolic and flavonoid contents observed in the 70% aqueous methanol extract can be attributed to the higher solubility of polar phenolic compounds in hydroalcoholic systems. This finding is consistent with the UHPLC-HRMS/MS results, which demonstrated a strong enrichment of phenolic acids and flavonoids—particularly chlorogenic acid and diosmetin—in the aqueous methanol extract. The relatively low values obtained for the *n*-hexane extract further support the limited extraction of polar compounds by nonpolar solvents. Considering the well-established role of phenolic compounds in antioxidant and enzyme inhibitory mechanisms, the elevated phenolic and flavonoid contents of the 70% aqueous methanol extract may significantly contribute to the antioxidant and α-amylase inhibitory activities observed in the present study. In particular, the high abundance of chlorogenic acid, diosmetin, and related flavonoids identified by UHPLC-HRMS/MS analysis supports the biochemical basis of the observed bioactivities.

### 3.8. In Vitro Biological Activity Results

The evaluation of antioxidant activity was conducted through a series of in vitro assays, including DPPH, ABTS, CUPRAC, FRAP, ORAC, and β-carotene bleaching. The inhibitory potential of α-amylase was also evaluated. The results from the ABTS, CUPRAC, FRAP, and ORAC tests were shown as Trolox equivalents, while the results for DPPH, β-carotene bleaching, and α-amylase inhibition were reported as IC_50_ values (µg/mL). Table 9 summarizes the results of all biological activities.

With an IC_50_ value of 0.043 ± 0.002 mg/mL, the present study revealed that the 70% aqueous methanol extract produced from *O. onur-koyuncui* seeds demonstrated markedly stronger antioxidant activity than previously recorded values reported for other *Onosma* species. With several solvents employed for extraction, antioxidant studies in *Onosma* species have mostly concentrated on aerial parts. In previous studies including *O. bulbotrichum*, *O. riedliana*, *O. sericea*, *O. stenoloba*, and *O. isaurica*, DPPH radical scavenging capabilities (reported as IC_50_) were found within a range of 2.19 to 8.03 mg/mL [5,11,181].

In the present study, the FRAP value of the 70% aqueous methanol extract from *O. onur-koyuncui* seeds was calculated as 255.6 mg TE/g_extract_ (1.021 µmol Trolox/mg_extract_), exceeding the previously reported values [11,181,192]. The *n*-hexane extract exhibited a FRAP value of 90.88 mg TE/g_extract_ (0.363 µmol Trolox/mg_extract_), falling within the range reported for non-seed extracts of other *Onosma* species. To the best of our knowledge, this study provides the first FRAP evaluation conducted on *Onosma* seeds. In previous reports on *Onosma* species, the FRAP values of extracts obtained from aerial parts or roots varied between 36.87 and 230.27 mg TE/g_extract_, depending on the extraction solvent and plant part. However, these studies focused exclusively on non-seed parts.

The CUPRAC values obtained in this study for *O. onur-koyuncui* seeds were 0.0033 µmol Trolox/mg_extract_ for the 70% aqueous methanol extract and 0.0003 µmol Trolox/mg_extract_ for the *n*-hexane extract, indicating a higher reducing capacity for the polar extract. To the best of our knowledge, this study represents the first CUPRAC evaluation performed on *Onosma* species. Previous studies on *Onosma* species have primarily focused on aerial parts and reported CUPRAC values using different units, such as µmol TE/g_dry plant_ or mg TE/g_extract_. For instance, aqueous extract of *O. gigantea* exhibited CUPRAC values ranging from 0.86 ± 0.01 to 50.23 ± 0.19 μmol TE/g_dry plant_ [183], while methanolic extracts of *O. riedliana*, *O. sericea* and *O. stenoloba* showed values ranging from 142.88 ± 1.49 mg TE/g_extract_ and with 380.28 ± 2.43 mg TE/g_extract_ [181]. In addition, some studies have reported antioxidant capacities of *Onosma* species using IC_50_ values expressed in mg/mL, such as for the methanolic extracts of the aerial parts of *O. isaurica* and *O. bracteosa* (1.91 ± 0.03 and 1.62 ± 0.01 mg/mL, respectively) [192]. However, these values are derived from different assay principles and are therefore not directly comparable to CUPRAC results. In the present study, CUPRAC values were expressed as µmol Trolox equivalents per mg extract to ensure consistency across antioxidant assays. Therefore, comparisons with previously reported data should be interpreted with caution because of differences in unit expression, assay type, and experimental conditions.

This study presents the first TEAC assessment for *Onosma* seeds. The 70% aqueous methanol extract of *O. onur-koyuncui* seeds demonstrated a TEAC value of 0.0033 µmol Trolox/mg_extract_, while the *n*-hexane extract exhibited lower activity of 0.0003 µmol Trolox/mg_extract_. Previous research has reported TEAC values for *Onosma* species using different units, depending on species, plant parts, and extraction solvents. TEAC values for *O. gigantea* have been reported in the range of approximately 32 and 134 µmol TE/g [182], whereas other studies have expressed antioxidant activity as IC_50_ values in mg/mL, reflecting differences in assay principles [194]. As described for the CUPRAC assay, TEAC values in the present study were expressed as µmol Trolox equivalents per mg extract to maintain consistency across antioxidant assays. Therefore, comparisons with literature data should be interpreted with caution, as differences in unit expression and experimental conditions may affect the reported results.

ORAC data have not yet been reported for any *Onosma* species in the current literature. To the best of our knowledge, the present study provides the first ORAC evaluation reported for both *O. onur-koyuncui* seeds and the genus *Onosma*. The *n*-hexane extract exhibited an ORAC value of 0.008 ± 0.000 µmol Trolox/mgextract, whereas the 70% aqueous methanol extract showed 0.002 ± 0.000 µmol Trolox/mg_extract_. Similarly, no previous β-carotene bleaching assay data are currently available from the β-carotene bleaching assay for *Onosma* species. Strong lipid peroxidation inhibitory action was shown in the present work by the 70% aqueous methanol extract of *O. onur-koyuncui* seeds with an IC_50_ value of 0.381 ± 0.011 mg/mL. Though its IC_50_ value could not be determined, the *n*-hexane extract showed 7.692 ± 0.001% inhibition. To the best of our knowledge, this work presents *Onosma* seed’s first β-carotene bleaching activity data (Table 10).

In general, phenolic compounds are considered the major contributors to antioxidant activity. However, lipophilic constituents may also influence electron-transfer and radical-scavenging assays depending on assay conditions and extract composition. Several studies have demonstrated that nonpolar extracts enriched in unsaturated fatty acids, sterols, and other apolar phytochemical constituents can display measurable antioxidant responses in CUPRAC and ORAC assays [195,196,197]. Therefore, the comparatively notable activity observed for the *n*-hexane extract of *O. onur-koyuncui* seeds may be partly associated with its lipophilic antioxidant components rather than phenolic constituents alone.

No prior research has examined the α-amylase inhibitory properties of *Onosma* seeds. In this investigation, the 70% aqueous methanol extract of *O. onur-koyuncui* seeds showed notable α-amylase inhibitory activity (IC_50_ value: 0.40 ± 0.01 mg/mL). As expected, the reference inhibitor acarbose exhibited markedly stronger inhibition than the plant extracts, consistent with its lower IC_50_ value. Although the IC_50_ could not be estimated, the *n*-hexane extract showed a low inhibition rate of 30.42 ± 0.10% at the tested concentration. This study is the first to assess α-amylase inhibition in the seeds of *O. onur-koyuncui* and the *Onosma* genus (Table 9). Previous studies on *Onosma* species reported that *O. bulbotricha* DC. aerial parts showed α-amylase inhibition with IC_50_ values of 2.10 ± 0.04 mg/mL (ethyl acetate extract) and 2.82 ± 0.02 mg/mL (methanolic extracts) from *O. sericea* and *O. stenoloba* aerial parts inhibited α-amylase at 1.26 ± 0.07 mmol ACAE/g_extract_ and 0.89 ± 0.02 mmol ACAE/g_extract_ [11]. The methanolic extracts of *O. isaurica* and *O. bracteosa* aerial parts inhibited α-amylase with 130.19 ± 13.86 mg ACEs/g_extract_ and 396.02 ± 6.58 mg ACEs/g_extract_, respectively [192]. *O. inexspectata* and *O. armena* had IC_50_ values of 3.78 ± 0.34 mg/mL and 3.85 ± 0.34 mg/mL, respectively. *O. gigantea* inhibited α-amylase with 1.07–6.75 ACEs/g_plant material_ in methanol [182].

The antioxidant and α-amylase inhibitory activities observed for the 70% aqueous methanol extract occurred alongside its high phenolic and flavonoid contents and the presence of compounds such as chlorogenic acid, diosmetin, and catechol derivatives [172,187,198]. Similarly, the *n*-hexane extract contained unsaturated fatty acids and β-sitosterol, which have been associated with biological activities in previous studies [199,200,201]. However, the present extract-level data do not establish a causal relationship between individual constituents and the measured biological activities. The observed effects may reflect additive, synergistic, or antagonistic interactions among multiple constituents within these complex phytochemical mixtures. Therefore, the contribution of individual metabolites cannot be determined without further bioactivity-guided fractionation and evaluation of isolated compounds, individually and in combination. The use of multiple complementary antioxidant assays nevertheless provides a broader assessment of extract-level antioxidant behavior by evaluating different mechanisms, including radical scavenging, reducing capacity, and inhibition of lipid peroxidation [202,203].

## 4. Conclusions

This study provides the first comprehensive seed-focused phytochemical and biological characterization within the genus *Onosma*, establishing baseline information on the volatile, fatty acid, mineral, and polar and lipophilic phytochemical composition of *O. onur-koyuncui* seeds. The complementary analytical approaches revealed a chemically diverse seed profile, with phenolic acids and flavonoids predominating in the 70% aqueous methanol extract and fatty acids, phytosterols, and other lipophilic constituents characterizing the *n*-hexane extract. The study also provides the first volatile and mineral composition data for seeds within the genus, thereby addressing an important gap in the phytochemical knowledge of *Onosma*.

The extracts exhibited antioxidant and α-amylase inhibitory activities, with the 70% aqueous methanol extract generally showing stronger responses. However, these extract-level biological effects cannot be attributed to individual constituents without further fractionation and compound-level evaluation, as additive, synergistic, or antagonistic interactions among multiple constituents may contribute to the observed activities. Overall, the findings expand current knowledge of the seed chemistry of *Onosma* and demonstrate the value of seed-focused phytochemical investigation in this underexplored genus.

The findings should be interpreted within the sampling and extraction framework of the present study. *O. onur-koyuncui* is a local endemic species with a highly restricted known natural distribution; therefore, sampling from geographically distinct populations was not applicable within its known distribution. Nevertheless, as the seeds were collected during a single maturation period, potential temporal variation in their phytochemical composition could not be assessed. The present study was designed as a phytochemical and bioactivity characterization rather than an agronomic, technological, or economic assessment. Therefore, cultivation feasibility, seed productivity, scalability, availability, and economic viability were not evaluated, and practical utilization cannot be established from the present data. In addition, the substantial polarity difference between *n*-hexane and 70% aqueous methanol should be considered when interpreting the observed compositional differences. These solvents were selected to provide complementary coverage of lipophilic and polar constituents rather than as directly comparable extraction systems. Future studies should evaluate seed material collected across different years and investigate greener extraction systems, including solvents of intermediate polarity, to provide broader coverage across the polarity range while improving extraction sustainability.

## Figures and Tables

**Table 1:** Fatty acid methyl esters (FAMEs) composition of *O. onur-koyuncui* seeds.

No	RRI	Compound	(%)
1	2016	Methyl myristate [=Tetradecanoic acid methyl ester]; (14:0)	tr
2	2125	Methyl pentadecanoate [=Pentadecylic acid methyl ester]; (15:0)	tr
3	2223	Methyl palmitate [=Hexadecanoic acid methyl ester]; (16:0)	7.61 ± 0.11
4	2251	Methyl palmitoleate [=*(Z)*-9-hexadecenoic acid methyl ester]; (16:1); ω-7	0.09 ± 0.00
5	2330	Methyl heptadecanoate [=Margaric acid methyl ester]; (17:0)	0.08 ± 0.00
6	2436	Methyl stearate [=Octadecanoic acid methyl ester];(18:0)	3.33 ± 0.06
7	2468	Methyl oleate [=*(Z)-*9-Octadecenoic acid methyl ester]; (18:1); ω-9	18.24 ± 0.07
8	2469	Methyl elaidate [=*(E)-*9-Octadecenoic acid methyl ester]; (18:1); ω-9	0.89 ± 0.00
9	2509	Methyl linoleate [=*(Z,Z)-*9,12-Octadecadienoic acid methyl ester]; (18:2); ω-6	23.01 ± 0.13
10	2542	Methyl nonadecanoate [=Nonadecanoic acid methyl ester]; (19:0)	0.62 ± 0.00
11	2547	Methyl octadecatrienoate [=Octadecatrienoic acid methyl ester^#^]; (18:3); ω-6	9.06 ± 0.12
12	2572	Methyl octadecatrienoate [=Octadecatrienoic acid methyl ester^#^]; (18:3); ω-3	27.77 ± 0.08
13	2597	Methyl stearidonate [=*(Z,Z,Z,Z)-*6,9,12,15-Octadecatetraenoic acid methyl ester]; (18:4); ω-3	8.10 ± 0.03
14	2642	Methyl arachidate [=Eicosanoic acid methyl ester]; (20:0)	0.14 ± 0.00
15	2685	*(Z)-*11-Methyl eicosenoate [=Gondoic acid methyl ester]; *(20:1);* ω-9	0.83 ± 0.00
16	2843	Methyl behenate [=Docosanoic acid methyl ester]; (22:0)	0.07 ± 0.00
17	2868	Methyl erucate [=*(Z)*-13-Docosenoic acid methyl ester]; (22:1); ω-9	0.16 ± 0.00
18	3255	Methyl hexacosanoate [=Cerotic acid methyl ester]; (26:0)	tr
**Total**			**100.0**
Total ω-3			35.87
Total ω-6			32.1
Total ω-7			0.1
Total ω-9			20.1
Total unsaturated fatty acids			88.2

**Table 2:** Volatile compounds of *O. onur-koyuncui* seeds.

No	RRI_lit._	RRI_exp._	Compound	(%)	References
1	1032	1032	α-Pinene	1.01 ± 0.00	[68]
2	1076	1076	Camphene	0.27 ± 0.01	[69]
3	1093	1093	Hexanal	0.62 ± 0.01	[70]
4	1118	1118	β-Pinene	8.07 ± 0.08	[71]
5	1159	1159	δ-3-Carene	1.00 ± 0.02	[72]
6	1188	1188	α-Terpinene	1.86 ± 0.01	[71]
7	1200	1203	Limonene	1.23 ± 0.05	[73]
8	1232	1232	(*E*)-2-Hexenal	3.03 ± 0.04	[74]
9	1253	1255	γ-Terpinene	15.15 ± 0.03	[75]
10	1262	1266	(*E*)-β-Ocimene	2.32 ± 0.08	[75]
11	1280	1280	*p*-Cymene	15.00 ± 0.26	[76]
12	1351	1360	Hexanol	1.17 ± 0.03	[77]
13	1392	1400	Nonanal	2.76 ± 0.02	[77]
14	1441	1441	(*E*)-2-Octenal	0.52 ± 0.01	[78]
15	1452	1452	1-Octen-3-ol	0.37 ± 0.01	[79]
16	1463	1463	Heptanol	0.28 ± 0.00	[77]
17	1477	1475	Acetic acid	2.22 ± 0.03	[80]
18	1479	1479	(*E,Z*)-2,4-Heptadienal	0.31 ± 0.01	[81]
19	1495	1497	2-Ethyl hexanol	0.45 ± 0.00	[82]
20	1499	1506	Decanal	0.72 ± 0.01	[83]
21	1506	1507	(*E,E*)-2,4-Heptadienal	1.37 ± 0.05	[84]
22	1529	1523	(*E,Z*)-3,5-Octadien-2-one	2.23 ± 0.08	[85]
23	1541	1541	Benzaldehyde	2.41 ± 0.01	[83]
24	1548	1548	(*E*)-2-Nonenal	0.28 ± 0.00	[86]
25	1559	1562	Octanol	1.77 ± 0.02	[83]
26	1583	1583	*cis*-Isopulegone	0.33 ± 0.00	[87]
27	1590	1585	(*E,E*)-3,5-Octadien-2-one	1.41 ± 0.05	[85]
28	1604	1606	6-Methyl-3,5-heptadien-2-one	0.29 ± 0.00	[78]
29	1648	1638	β-Cyclocitral	0.28 ± 0.00	[83]
30	1656	1655	(*E*)-2-Decenal	0.43 ± 0.00	[88]
31	1656	1660	Benzene acetaldehyde	8.44 ± 0.09	[88]
32	1768	1764	(*E*)-2-Undecenal	0.51 ± 0.00	[83]
33	1798	1798	Methyl salicylate	1.10 ± 0.03	[71]
34	1802	1802	Cuminaldehyde	9.62 ± 0.07	[89]
35	1811	1811	*p*-Mentha-1,3-dien-7-al	0.36 ± 0.00	[89]
36	-	1815	*p*-Menta-1,4-dien-7-al	1.27 ± 0.08	-
37	1825	1827	(*E,E*)-2,4-Decadienal	0.55 ± 0.00	[83]
38	1862	1860	Hexanoic acid (=Caproic acid)	1.52 ± 0.06	[90]
39	1862	1868	(*E*)-Geranylacetone	0.30 ± 0.00	[91]
40	-	1890	2,2,4-Trimethyl-3-carboxyisopropyl-isobutyl pentanoate ^#^	0.11 ± 0.00	-
41	-	1957	Benzene acetonitril ^#^	0.09 ± 0.00	-
42	1936	1958	(*E*)-β-Ionone	0.33 ± 0.01	[77]
43	2004	2004	Phenol	0.24 ± 0.00	[92]
44	2045	2039	Isopropyl myristate	0.12 ± 0.01	[89]
45	2052	2053	Anisaldehyde	0.39 ± 0.00	[93]
46	2051	2057	Octanoic acid (=Caprylic acid)	0.48 ± 0.00	[90]
47	2131	2131	Hexahydrofarnesyl acetone	0.11 ± 0.00	[94]
48	2152	2153	Nonanoic acid (=Pelargonic acid)	0.73 ± 0.01	[90]
49	2195	2191	4-Ethylphenol	0.19 ± 0.00	[95]
50	2218	2218	4-Vinyl guaiacol	0.10 ± 0.00	[96]
51	2239	2239	Carvacrol	0.10 ± 0.00	[97]
52	2252	2255	Isopropyl palmitate	0.11 ± 0.00	[89]
53	2298	2298	Decanoic acid (=Capric acid)	0.19 ± 0.00	[98]
54	-	2318	Methyl propyl salicylate ^#^	0.28 ± 0.01	-
55	2931	2931	Hexadecanoic acid (=Palmitic acid)	0.79 ± 0.01	[99]
**Total**					**97.32**

**RRI_exp_**.: Experimentally calculated retention index against *n*-alkanes for polar column; **RRI_lit_**_.:_ Retention index from literature **#**: It is identified by the Wiley-NIST library spectrum similarity; **%**: calculated from FID data.

**Table 3:** Mineral composition of *O. onur-koyuncui* seeds analyzed by ICP-OES.

Mineral	mg/g_dw_	Mineral	mg/g_dw_
Magnesium	2.65 ± 0.02	Nickel	Nd
Sodium	0.25 ± 0.00	Iron	0.26 ± 0.00
Aluminum	0.25 ± 0.00	Copper	Nd
Zinc	0.05 ± 0.00	Calcium	28.50 ± 0.07
Titanium	Nd	Potassium	5.90 ± 0.03

Nd: Not Detected. Nickel, copper, and titanium concentrations were below the method detection limits (Ni: 2 ppb, Cu: 2 ppb, Ti: 2 ppb); dw: dry weight.

**Table 4:** Extraction yields and extract amounts of *O. onur-koyuncui* seeds.

Extract Type	Amounts of Extract, g	Yield *, %
*n*-Hexane Extract	5.93	20.17
70% Aqueous Methanol Extract	18.11	3.79

* It was calculated based on the dry weight of the seed material.

**Table 5:** Chemical composition of the silylated extracts of *O. onur-koyuncui* seeds analyzed by GC-FID/MS.

No	RRI ^#^	Compound *	*n*-Hexane Extract	70% Aqueous Methanol Extract
			%	%
1	1506	Malic acid-3 TMS	-	0.29 ± 0.01
2	1791	α-Glycerophosphoric acid -4 TMS	-	0.28 ± 0.00
3	1843	α-Fructofuranose-5 TMS	0.27 ± 0.00	0.11 ± 0.00
4	1852	β-D-Fructofuranose-5 TMS	0.20 ± 0.00	0.10 ± 0.00
5	1858	Fructopyranose-5 TMS	0.16 ± 0.01	-
6	1885	α-Glucofuranose-5 TMS	-	0.21 ± 0.00
7	1898	Quinic acid-5 TMS	-	0.38 ± 0.01
8	1929	α-Glucopyranose-5 TMS	0.26 ± 0.00	0.46 ± 0.01
9	1939	β-Galactopyranosis-5 TMS	0.17 ± 0.00	-
10	1942	β-Mannopyranose-5 TMS	-	0.33 ± 0.01
11	2023	β -Glucopyranose-5 TMS	0.60 ± 0.01	0.63 ± 0.02
12	2049	Hexadecanoic acid (=Palmitic acid)-TMS	5.41 ± 0.09	1.01 ± 0.03
13	2093	Catechol lactate-4 TMS	-	12.90 ± 0.11
14	2151	*(E)-*Caffeic acid-6 TMS	-	2.31 ± 0.02
15	2158	Octadecan-1-ol-TMS	0.36 ± 0.00	-
16	2217	*(Z,Z)-*9,12-Octadecadienoic acid (=Linoleic acid)-TMS	8.10 ± 0.12	4.51 ± 0.02
17	2221	* (Z)- * 9-Octadecenoic acid (=Oleic acid)-TMS	14.86 ± 0.06	9.01 ± 0.02
18	2225	* (E)- * 9-Octadecenoic acid (=Elaidic acid)-TMS	1.10 ± 0.01	-
19	2250	Octadecanoic acid (=Stearic acid)-TMS	2.00 ± 0.07	0.64 ± 0.02
20	2413	11-Eicoceneic acid-TMS	0.28 ± 0.01	-
21	2448	Eicosanoic acid (=Arachidic acid)-TMS	0.36 ± 0.02	-
22	2500	Pentacosane	0.27 ± 0.00	-
23	2555	Docosan-1-ol (=Behenyl alcohol)-TMS	1.00 ± 0.01	-
24	2591	Sucrose derivative-I	-	2.46 ± 0.03
25	2600	Hexacosane	0.27 ± 0.00	-
26	2608	α-Glyceryl palmitate (=Monopalmitin)-2 TMS	0.41 ± 0.01	-
27	2646	Docosanoic acid (=Behenic acid)-TMS	0.44 ± 0.00	-
28	2650	Sucrose derivative-II	0.36 ± 0.00	10.54 ± 0.08
29	2700	Heptacosane	0.79 ± 0.01	-
30	2704	Sucrose derivative-III	0.20 ± 0.01	0.34 ± 0.01
31	2713	Sucrose derivative-IV	-	7.77 ± 0.05
32	2722	Sucrose derivative-V	-	1.74 ± 0.02
33	2735	Sucrose derivative-VI	0.36 ± 0.00	-
34	2737	Sucrose derivative-VII	0.18 ± 0.00	-
35	2739	Trichosanoic acid (=Tricosylic acid)-TMS	1.10 ± 0.07	-
36	2765	α -Glyceryl linoleate-2 TMS	1.35 ± 0.03	-
37	2769	α Glyceryl oleate-2 TMS	1.01 ± 0.08	-
38	2835	Squalene	0.89 ± 0.01	-
39	2844	Tetracosanoic acid (=Lignoceric acid)-TMS	0.67 ± 0.00	-
40	2900	Nonacosane	1.00 ± 0.07	-
41	2953	Hexacosan-1-ol (=Ceryl alcohol)-TMS	0.40 ± 0.01	-
42	3014	γ-Tocopherol-TMS	4.67 ± 0.02	-
43	3144	Heptacosanoic acid (=Carboceric acid)-TMS	0.20 ± 0.00	-
44	3184	*crypto*-Chlorogenic acid-6 TMS	-	3.81 ± 0.04
45	3264	Luteolin-3 TMS	-	0.90 ± 0.00
46	3275	Campesterol-TMS	10.37 ± 0.16	-
47	3305	Stigmasterol-TMS	1.06 ± 0.01	-
48	3368	β-Sitosterol-TMS	13.69 ± 0.13	0.40 ± 0.01
49	3378	Avenasterol-TMS	4.77 ± 0.02	-
50	3404	Sucrose derivative-VIII	0.20 ± 0.01	2.91 ± 0.02
51	3442	Sucrose derivative-IX	-	0.44 ± 0.00
52	3469	Rosmarinic acid-5 TMS	-	14.03 ± 0.17
53	3480	Sucrose derivative-X	-	3.55 ± 0.08
54	3502	Sucrose derivative-XI	-	1.83 ± 0.05
55	3585	Melezitose-11 TMS	-	7.77 ± 0.04
**Total**			**79.79**	**91.66**

RRI ^#^: Relative retention indices for apolar column; *: ≥0.5%.

**Table 6:** Identified and quantified phenolic compounds in the *n*-hexane extract of *O. onur-koyuncui* seeds.

Compound	RT	Mol. Formula	Exact Mass	*m*/*z* (Expected)	*m*/*z* (Apex)	Δmass (ppm)	MS/MS (MS^2^) Fragments	mg/g_dw_
Epicatechin	4.33	C_15_H_14_O_6_	289.0714	289.07176	289.07175	−0.04557	289.0364; 245.0132; 205.0366; 179.0111	0.64 ± 0.00
Taxifolin	5.01	C_15_H_12_O_7_	303.05095	303.05103	303.05093	−0.31731	57.03462; 125.02453; 151.00378; 175.04005; 285.04086	0.02 ± 0.00
Quercetin	6.65	C_15_H_10_O_7_	301.03438	301.03538	301.03506	−1.04739	65.0034; 107.01403; 121.02964; 151.00386; 178.99893	0.07 ± 0.00
Naringenin	6.79	C_15_H_12_O_5_	271.06065	271.0612	271.06137	0.63030	65.00339; 83.01398; 107.01394; 119.05033; 151.00385	0.03 ± 0.00
Diosmetin	7.36	C_16_H_12_O_6_	299.0561	299.0561	299.05588	−0.77683	284.033; 256.038; 151.003; 133.029, 107.014; 65.003	0.44 ± 0.02

**Table 7:** Identified and quantified phenolic compounds in the 70% aqueous methanol extract of *O. onur-koyuncui* seeds.

Compound	RT	Mol. Formula	Exact Mass	*m*/*z* (Expected)	*m*/*z* (Apex)	Δmass (ppm)	MS/MS (MS^2^) Fragments	mg/g_dw_
Protocatechuic acid	2.12	C_7_H_6_O_4_	153.0193	153.01933	153.0192	0.8786	109.0293; 108.0170	2.02 ± 0.01
4-Hydroxybenzoic acid	3.26	C_7_H_6_O_3_	137.02387	137.02442	137.02435	1.04556	65.0396; 93.03473; 137.02458	6.49 ± 0.27
Chlorogenic acid	3.83	C_16_H_18_O_9_	353.08781	353.08781	353.08759	0.63596	59.0139; 85.02957; 93.03461; 127.04014; 191.05635	51.29 ± 0.77
Caffeic acid	3.99	C_9_H_8_O_4_	179.03498	179.03498	179.0349	0.46443	89.03983; 107.05002; 134.03746; 135.04527; 179.03506	3.59 ± 0.03
*p*-Coumaric acid	4.79	C_9_H_8_O_3_	163.0395	163.04007	163.03999	0.47052	65.0397; 91.0554; 93.0346; 104.02666; 119.05025	1.31 ± 0.01
Taxifolin	5.02	C_15_H_12_O_7_	303.05830	303.05103	303.05109	0.01521	57.03458; 125.02444; 151.00366; 175.04013; 285.04068	6.59 ± 0.02
Naringin	5.80	C_27_H_32_O_14_	579.1719	579.17193	579.17188	0.09496	65.00337; 107.01395; 119.05031; 151.00381; 271.06158	0.08 ± 0.00
Salicylic acid	5.87	C_7_H_6_O_3_	137.0239	137.02442	137.02431	0.82284	65.0397; 93.03461; 137.02446	1.82 ± 0.01
Eriodictyol	6.27	C_15_H_12_O_6_	287.0558	287.05611	287.05624	0.46645	65.00333; 83.01392; 107.01386; 135.04526; 151.00374	0.99 ± 0.03
Abscisic acid	6.53	C_15_H_20_O_4_	263.12888	263.12888	263.12872	0.59612	122.03735; 153.09222; 203.10822; 204.11578; 219.13921	0.09 ± 0.00
Quercetin	6.63	C_15_H_10_O_7_	301.03438	301.03538	301.03534	0.13502	65.00333; 107.01396; 121.02949; 151.00371; 178.99849	4.17 ± 0.00
Naringenin	6.78	C_15_H_12_O_5_	271.06065	271.0612	271.06131	0.40513	65.00334; 83.01392; 107.01393; 119.05027; 151.00374	0.17 ± 0.00
Luteolin	6.90	C_15_H_10_O_6_	286.04046	285.04046	285.03989	2.01208	63.02406; 65.00332; 133.02953; 284.03284; 285.04071	2.10 ± 0.00
Diosmetin	7.34	C_16_H_12_O_6_	299.0561	299.05611	299.05594	0.57273	107.0138; 151.00352; 284.03284	42.95 ± 1.69

Δmass (ppm) is defined as the deviation of the observed precursor ion mass from the corresponding theoretical precursor ion mass ([M−H]^−^). Compounds were identified by comparison with authentic reference standards, as well as by matching retention times, accurate mass measurements, and MS/MS fragmentation patterns. All compounds listed in this table were quantified using external calibration curves constructed from authentic reference standards. Standard deviations reported as 0.00 indicate values below the reporting precision (two decimal places). dw: dry weight.

**Table 8:** Total phenolic and flavonoid contents of *n*-hexane and 70% aqueous methanol extracts of *O. onur-koyuncui* seeds.

Extract Type	Total Phenolic Content, mg GAE/g_extract_	Total Flavonoid Content, mg QE/g_extract_
*n*-Hexane Extract	13.72 ± 0.51	4.57 ± 0.24
70% Aqueous Methanol Extract	112.19 ± 4.98	51.42 ± 0.85

**Table 9:** Antioxidant activities of *n*-hexane and 70% aqueous methanol extracts of *O. onur-koyuncui* seeds.

Extract Type	DPPH ^a)^ IC_50_ (mg/mL)	FRAP ^c)^, µmol Trolox/mg_extract_	CUPRAC ^c)^, µmol Trolox/mg_extract_	TEAC ^c)^, µmol Trolox/mg_extract_	ORAC ^c)^, µmol Trolox/mg_extract_	β-Carotene Peroxidation Inhibition ^a)^, IC_50_ (mg/mL)
*n*-Hexane extract	0.585 ± 0.002 ^b)^	0.363 ± 0.005	0.0008 ± 0.0000	0.0003 ± 0.0000	0.008 ± 0.000	7.692 ± 0.001 ^d)^
70% Aqueous methanol extract	0.043 ± 0.002	1.021 ± 0.002	0.0003 ± 0.0000	0.0033 ± 0.0000	0.002 ± 0.000	0.381 ± 0.011
Gallic acid	0.002 ± 0.000	-	-	-	-	-
Ascorbic acid	0.014 ± 0.000	-	-	-	-	-
Butylated hydroxyanisole	0.118 ± 0.009	-	-	-	-	0.009 ± 0.000

^a)^: IC_50_ values represent half maximal inhibitory concentrations.; ^b,d)^: IC_50_ values could not be determined as 50% inhibition was not reached within the tested concentration range (5 mg/mL). ^c)^: FRAP, CUPRAC, TEAC, and ORAC values were determined at a fixed extract concentration of 0.1 mg/mL.

**Table 10:** α-Amylase inhibitory effects of *n*-hexane and 70% aqueous methanol extracts of *O. onur-koyuncui* seeds.

Extract Type	α-Amylase Inhibition ^a)^, IC_50_ (mg/mL)
*n*-Hexane extract	30.42 ± 0.10 ^b)^
70% Aqueous methanol extract	0.40 ± 0.01
Acarbose	0.01 ± 0.00

^a)^: IC_50_ values represent half maximal inhibitory concentrations. ^b)^: IC_50_ value could not be determined as 50% inhibition was not reached within the tested concentration range (5 mg/mL).

## Data Availability

The datasets generated and analyzed during the current study are publicly available in the Zenodo repository at https://doi.org/10.5281/zenodo.20527370 and https://doi.org/10.5281/zenodo.20732531.

## Supplementary Materials

The supplementary analytical datasets supporting this study are publicly available in the Zenodo repository at: https://doi.org/10.5281/zenodo.20527370. In addition, the raw and processed UHPLC-HRMS/MS Orbitrap datasets supporting the identification, annotation, and quantification of phenolic compounds reported in Table 6 and Table 7 are publicly available in Zenodo at: https://doi.org/10.5281/zenodo.20732531. Figure S1: UHPLC-HRMS/MS Orbitrap total ion chromatogram (TIC) of the *n*-hexane extract of *O. onur-koyuncui* seeds. The annotated compounds were identified using retention times (RT), accurate mass measurements, authentic reference standards, and MS/MS fragmentation patterns.; Figure S2: UHPLC-HRMS/MS Orbitrap total ion chromatogram (TIC) of the 70% aqueous methanol extract of *O. onur-koyuncui* seeds. The annotated compounds were identified using retention times (RT), accurate mass measurements, authentic reference standards, and MS/MS fragmentation patterns; Figure S3: UHPLC-HRMS/MS Orbitrap extracted ion chromatograms (EICs) and corresponding MS^2^ spectra of the phenolic compounds identified in the *n*-hexane extract of *O. onur-koyuncui* seeds. Compound annotations were supported by retention times (RT), precursor ions, accurate mass measurements, and MS/MS fragmentation patterns. The chromatograms and spectra include RT, precursor-ion, and fragment-ion information supporting compound annotation and correspond to the compounds listed in Table 6. Figure S4: UHPLC-HRMS/MS Orbitrap extracted ion chromatograms (EICs) and corresponding MS^2^ spectra of the phenolic compounds identified in the 70% aqueous methanol extract of *O. onur-koyuncui* seeds. The chromatograms and spectra provide RT, precursor-ion, and fragment-ion information supporting compound identification. The chromatograms and spectra include RT, precursor-ion, and fragment-ion information supporting compound annotation and correspond to the compounds listed in Table 7; Table S5: Calibration curve parameters, regression equations, and coefficients of determination (R^2^) of phenolic reference standards used for UHPLC-HRMS/MS quantitative determination of the compounds identified in Table 6 and Table 7.
